# Evolution of the electrochemical interface in sodium ion batteries with ether electrolytes

**DOI:** 10.1038/s41467-019-08506-5

**Published:** 2019-02-13

**Authors:** Kaikai Li, Jun Zhang, Dongmei Lin, Da-Wei Wang, Baohua Li, Wei Lv, Sheng Sun, Yan-Bing He, Feiyu Kang, Quan-Hong Yang, Limin Zhou, Tong-Yi Zhang

**Affiliations:** 1 Shenzhen Environmental Science and New Energy Technology Engineering Laboratory, Tsinghua-Berkeley Shenzhen Institute (TBSI), Tsinghua University, Shenzhen, 518055 China; 2 Interdisciplinary Division of Aeronautical and Aviation Engineering, The Hong Kong Polytechnic University, Hong Kong, China; 3 Department of Mechanical Engineering, The Hong Kong Polytechnic University, Hong Kong, China; 4 School of Chemical Engineering, The University of New South Wales, Sydney, 2052 NSW Australia; 5 Shenzhen Key Laboratory for Graphene-based materials and Engineering Laboratory for Functionalized Carbon Materials, Graduate School at Shenzhen, Tsinghua University, Shenzhen, 518055 China; 6 Materials Genome Institute, Shanghai University, 333 Nanchen Road, 200444 Shanghai, China; 7 Nanoyang Group, State Key Laboratory of Chemical Engineering, School of Chemical Engineering and Technology, Tianjin University, 300072 Tianjin, China

## Abstract

Ether based electrolytes have surfaced as alternatives to conventional carbonates allowing for enhanced electrochemical performance of sodium-ion batteries; however, the primary source of the improvement remains poorly understood. Here we show that coupling titanium dioxide and other anode materials with diglyme does enable higher efficiency and reversible capacity than those for the combination involving ester electrolytes. Importantly, the electrolyte dependent performance is revealed to be the result of the different structural evolution induced by a varied sodiation depth. A suit of characterizations show that the energy barrier to charge transfer at the interface between electrolyte and electrode is the factor that dominates the interfacial electrochemical characteristics and therefore the energy storage properties. Our study proposes a reliable parameter to assess the intricate sodiation dynamics in sodium-ion batteries and could guide the design of aprotic electrolytes for next generation rechargeable batteries.

## Abstract (web summary)

Sodium ion batteries are known to benefit from the use of ether electrolytes. Here the authors reveal the origin showing that the energy barrier of charge transfer at the electrolyte/electrode interface dominates the interfacial electrochemical characteristics and is favorably small.

## Introduction

Sodium-ion batteries (SIBs) have attracted more attention in recent years particularly for large-scale energy storage due to the natural abundance of sodium compared to lithium^1,2^. However, their performance including specific capacity, rate capability, and cycle life are severely hindered by the larger diameter of sodium ions than lithium ions^3,4^, and as a result there have been extensive efforts in searching for suitable electrode materials, in particular on the anode side^5,6^. In order to improve the electrochemical performance of the present anode materials, modifying the electrolyte is an important and effective approach. Most commonly used non-aqueous electrolytes inevitably decompose forming a solid electrolyte interphase (SEI) on the anode materials because the operating voltage is lower than their stable voltage range. Conventional electrolytes in SIBs are formulated using ester-based solvents similar to those used in lithium-ion batteries (LIBs)^7,8^, and these produce SEIs that are often reported to be not sufficiently stable and likely to cause severe polarization^9^. In this context, ether-based electrolytes are believed to be less useful in LIBs owing to inferior passivation on both anodes and cathodes, but they have recently been revived in SIBs because of their ability to trigger the highly reversible co-intercalation of sodium ions with an ether solvent into graphite^10–14^. The improved sodium storage performance has been observed on different anode materials like CuS nanosheets^3^, ZnS nanospheres^11^, bismuth^15^, and reduced graphene oxide (rGO)^9^ when ester-based electrolytes are replaced with ether analogs. It is proposed that this improvement is due to the suppression of dissolved polysulfide intermediates^3^, faster Na^+^ transport, good electrode wettability^11,15^, and the formation of a thinner SEI^9^. Su et al.^11^ proved that Na ions can be easily absorbed on an ether-based solvent and there is a low energy barrier for Na ion diffusion in it, and Seh et al.^16^ proved that a glyme-based electrolyte (a typical type of ether-based electrolyte) is superior to an EC/DEC-based electrolyte (a typical ester-based electrolyte; here ethylene carbonate is denoted as EC and diethyl carbonate is denoted as DEC) for a Na metal anode due to the formation of low resistivity SEI, making a large advance on the research of ether-based electrolytes for SIBs. However, the interfacial electrochemical characteristics, especially charge transfer in different types of electrolyte has not yet been systematically investigated. A deeper understanding of the issue is urgently needed so as to clarify the intrinsic origin of the improvement produced by ether-based electrolytes in SIBs. This is a key point in the revival of this technology using an ether-based electrolyte^17,18^.

Selecting a typical and ideal anode material is crucial to address this issue and TiO_2_ is preferably chosen in this study considering the following points. Firstly, the sodiation of TiO_2_ results in the formation of an amorphous sodium titanate phase so that the structural evolution during sodiation can be easily detected^19–22^. Secondly, the intercalation mechanism and the small volume change produced by pseudocapacitive sodium storage in amorphous sodium titanate^23,24^ also guarantee the formation of a mechanically stable interface between electrolyte and electrode, which is difficult to achieve in other anodes that involve large volume changes or conversion reactions upon sodiation. Thirdly, unlike the long flat plateau at ~ 1.75 V vs Li/Li^+^ in lithium-ion batteries (LIBs), the operating potential of TiO_2_ in SIBs decreases to 0.005 V, which inevitably results in the formation of SEI layer on the electrode^25^. Lastly, optimizing electrolyte is also promising to address the bottleneck of practical application of TiO_2_ anodes including low initial coulombic efficiency (ICE) and fast capacity decay^18,26–28^, although current efforts have mostly targeted the improvement of the electronic conductivity in TiO_2_^29–33,^^34–36^^,37^. Further extending the knowledge and experience of above TiO_2_-based research to other metal and carbon-based anodes can better illustrate the generality and origin of ether-enhanced interfacial electrochemical characteristics.

In the present work, we investigate ether-induced interfacial electrochemical characteristics for SIBs by coupling anatase TiO_2_ with a diglyme-based electrolyte. Such a TiO_2_ anode shows excellent capacity retention and rate capability, compared to those coupled with an EC/DEC-based electrolyte. Insights into the structural evolution and sodiation dynamics obtained by in operando Raman, X-ray diffraction (XRD), and electrochemical kinetic studies reveal that the charge transfer characteristics of the electrolyte/electrode interface play a vital role in determining the performance, which is also confirmed for Sn, rGO, and CMK-3 anodes. Comprehensive X-ray photoelectron spectroscopy (XPS) analyses show that the SEI formed in different electrolytes is composed of different organic species and exhibits distinct composition changes along the SEI depth with more inorganic species in the inner region when formed in an ether-based electrolyte. This is the origin of the electrolyte-dependent sodiation dynamics and battery performance. Our results indicate a general and fundamental explanation of the improved electrochemical performance of different anodes for SIBs induced using an ether-based electrolyte. This study might suggest an avenue for the design of efficient electrolyte/electrode interfaces for improved performance of non-aqueous rechargeable batteries.

## Results

### Sodium ion storage performance

**Fig. 1:**
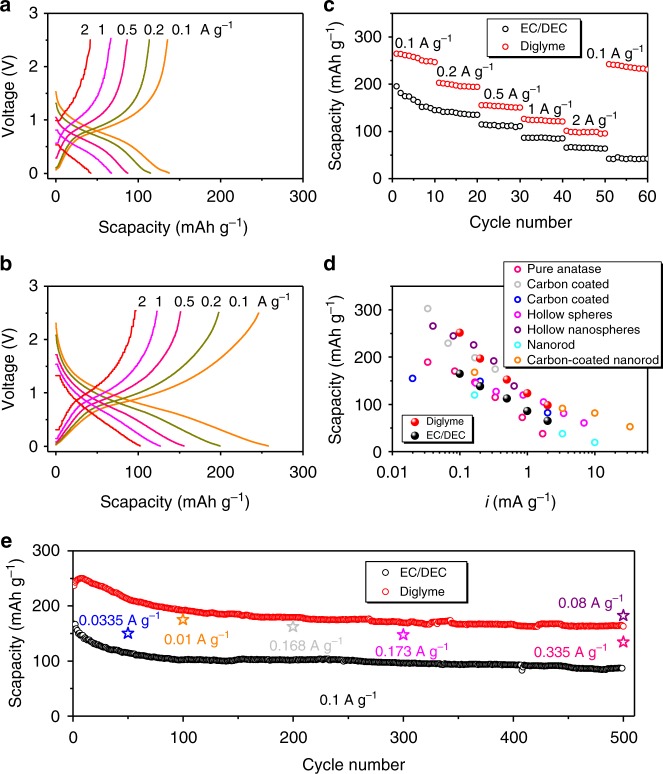
Battery performance. **a** Voltage profiles of the TiO_2_ anode at different rates using the EC/DEC-based electrolyte (scapacity: specific capacity). **b** Voltage profiles of the TiO_2_ anode at different rates using the diglyme-based electrolyte. Note that the profiles at 0.1 A g^−1^ in **a** and **b** are for the third cycle while the others are for the first cycle at corresponding rates. **c** Rate performance. **d** Performance compared with anatase TiO_2_-based SIB anodes reported in the literature, i.e., pure anatase TiO_2_^56^, carbon-coated anatase^57,58^, hollow spheres^23^ and hollow nanospheres^59^, anatase nanorods^60^, and coated anatase nanorods^60^. **e** Cyclic performance at 0.1 A g^−1^, and comparison with cyclic performance reported in the literature

Rhombic anatase TiO_2_ nanocrystals were synthesized using a solvothermal technique similar to that reported elsewhere^38^. The average size of the nanocrystals was around 16 nm as shown in Supplementary Figures 1 and 2. Supplementary Figure 2b is a HRTEM image of an individual TiO_2_ nanocrystal, clearly showing the TiO_2_ anatase crystal lattice with (101) planes and a *d*-spacing of 0.35 nm. The XRD pattern and Raman spectrum (Supplementary Figure 2c and d) confirm that the anatase TiO_2_ nanocrystals are monophasic (JCPDS 21-1272) with a high crystallinity. The SIB anode was prepared using these TiO_2_ nanocrystals (70 wt%) as the active material, carbon black (15 wt%) as the conductive agent, and polyvinylidene fluoride (PVDF) (15 wt%) as the binder. Coin cells were then assembled using two different solvent-based electrolytes, i.e., NaCF_3_SO_3_ dissolved in EC/DEC and NaCF_3_SO_3_ in diglyme. Figure 1a shows the discharge/charge voltage profiles of SIBs prepared with this TiO_2_ anode and the EC/DEC electrolyte, measured at current densities ranging from 0.1 to 2 A g^−1^. The voltage profiles are linear with no obvious plateau regions, which is different from the flat and prolonged voltage plateau observed in LIBs^39^. This indicates that the sodiation of TiO_2_ proceeds by a different mechanism from lithiation, which might indicate a single-phase Na-intercalation process^40^. As clearly demonstrated by others, the sodiation of anatase TiO_2_ is a solid-solution-like reaction^21,41^. The operation potential of these anatase nanocrystals as the SIB anode is averaged to be ~0.8 V, which is significantly lower than that for Li intercalation (~1.75 V vs. Li/Li^+^)^39^, making TiO_2_ more promising for practical applications. When EC/DEC is replaced by diglyme, a significant improvement is observed in the anode performance, as shown in Fig. 1b. For instance, the specific capacity at 0.2 A g^−1^ is improved from 114.8 to 199.4 mAh g^−1^. Increasing the current rate from 0.1 to 2 A g^−1^ causes a capacity loss in both cases, but it is more severe in the EC/DEC case (decreasing from 137.5 to 42.2 mAh g^−1^) than in diglyme (decreasing from 257.9 to 102.1 mAh g^−1^). It is also worth noting that using the diglyme-based electrolyte obviously improves the ICE of TiO_2_ as an SIBs anode, which is one of the critical issues for TiO_2_ SIB anodes. As shown in Supplementary Figure 3, the ICE is as high as 69% in a diglyme-based electrolyte, much higher than the 33% in an EC/DEC-based electrolyte, although a further increase is still needed for the real applications.

Figure 1c, d shows the rate capabilities of the TiO_2_ anode using two different electrolytes. The cells using the diglyme-based electrolyte show an exceptional high rate capability, much higher than that for the EC/DEC system. Remarkable reversible capacities of ~252, 197, 153, 124, and 99 mAh g^−1^ were recorded for the diglyme case at current rates of 0.1, 0.2, 0.5, 1, and 2 A g^−1^, respectively. The corresponding values for EC/DEC are only ~165, 139, 113, 86, and 66 mAh g^−1^. When the current rate is decreased to 0.1 A g^−1^, the cell with diglyme retains its high capacity while the cell based on EC/DEC does not, indicating better reversibility when the diglyme-based electrolyte is used. Compared to the anatase TiO_2_ SIB anodes reported in the literature, the performance of the present TiO_2_ anode coupled with diglyme is among the best available. It is worth noting that the present TiO_2_ active materials are not carbon-modified or doped with other elements, implying a potential for further optimization by compositing with carbons or other elements. Both cells using diglyme and EC/DEC electrolytes have a quite stable long-life cyclic performance at 0.1 A g^−1^ as shown in Fig. 1e. But the absolute capacities of the cells using EC/DEC are much lower than those with the diglyme counterpart. After 500 cycles at 0.1 A g^−1^, a reversible capacity of 165 mAh g^−1^ was maintained for the cell based on the diglyme electrolyte, while the value was only 87 mAh g^−1^ for the case with EC/DEC. Our previous work^9^ demonstrated that carbon anodes such as rGO and CMK-3 showed much better efficiency and reversible capacity in the diglyme-based electrolyte than in EC/DEC system. Here, we further studied an Sn anode and found that the efficiency and reversible capacity in a diglyme-based electrolyte were superior to what was observed for EC/DEC (Supplementary Figure 4).

### Structural analysis

**Fig. 2:**
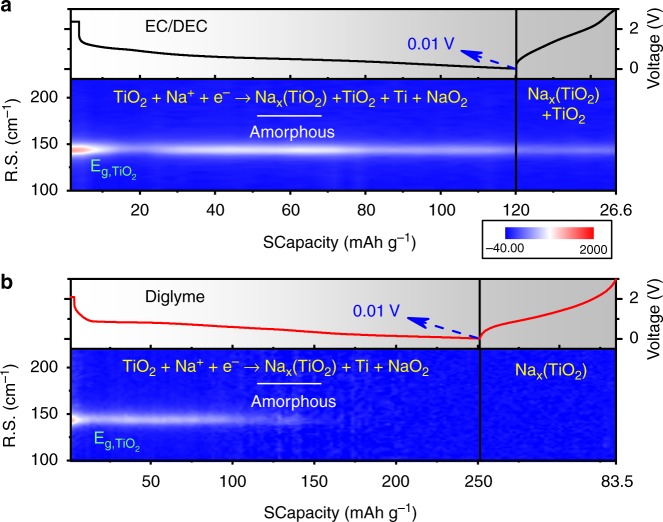
In operando Raman spectroscopy. Raman spectra of the TiO_2_ anode during the first cycle of electrochemical sodiation/de-sodiation at a rate of 100 mA g^−1^ (R.S.: Raman shifts), **a** using the EC/DEC-based electrolyte and **b** using the diglyme-based electrolyte^21^

To gain an insight into the excellent performance from the diglyme-based electrolyte and particularly into the Na-ion diffusion dynamics during sodiation/de-sodiation, we conducted in operando Raman spectroscopy (Supplementary Figure 5) and XRD (Supplementary Figure 8), and ex situ XRD to reveal the structural evolution, and scanned rate-dependent cyclic voltammetry (CV) as well as temperature-dependent electrochemical impedance spectroscopy (EIS). The sodiation of anatase TiO_2_ is an intercalation reaction described as follows: the sodium is intercalated into the TiO_2_ lattice, distorts the lattice, and partially reduces the TiO_2_ to form metallic titanium, sodium oxide, and amorphous sodium titanate^21^, which acts as the host for the Na ion storage in the following cycles. Taking the above mechanism into consideration, complete sodiation of TiO_2_ results in the disappearance of the Raman bands of crystalline TiO_2_, which will not reappear after de-sodiation. As shown in Fig. 2b, the Raman spectra evolution characteristics of the TiO_2_ anode coupled with a diglyme-based electrolyte during the first cycle conform to this mechanism. In sharp contrast, when using the EC/DEC-based electrolyte, the TiO_2_ cannot be fully sodiated even when the voltage is 0.01 V in the first cycle, as shown in Fig. 2a where the Raman signal of TiO_2_ can still be observed, indicating a lower accessible capacity than when using diglyme in the first cycle, and only after ~4 cycles is the TiO_2_ completely transformed to the amorphous phase (Supplementary Figure 6). This incomplete sodiation of anatase TiO_2_ has also been observed in an EC/PC (propylene carbonate)-based electrolyte^42^. The ex situ XRD results (Supplementary Figure 7) further prove the amorphization of the TiO_2_ during the first discharge in the diglyme-based electrolyte and the incomplete sodiation in the EC/DEC-based electrolyte. Ex situ XRD results of the Sn anode (Supplementary Figure 10) show the formation of the Na_*x*_Sn phase when discharged to 0.1 V in the diglyme-based electrolyte, which is not observed for the EC/DEC case. The in operando Raman spectroscopy and XRD (Supplementary Figure 9) along with the ex situ XRD results show that the sodiation of TiO_2_ and Sn is much easier in the diglyme-based electrolyte than in the EC/DEC-based electrolyte. In our previous work^9^, we showed that carbon materials like rGO and CMK-3 can be sodiated to a higher extent in the diglyme-based electrolyte than in the EC/DEC-based electrolyte.

### Dynamic analysis

**Fig. 3:**
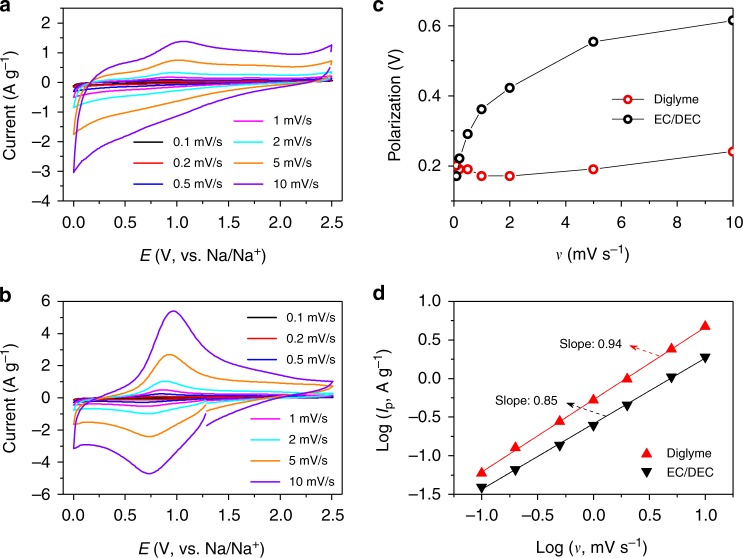
Scan rate-dependent cyclic voltammetry (CV) from the 3-electrode tests (see the experimental setup in Supplementary Figure 12). **a**, **b** CV curves of the TiO_2_ anode at different scan rates with the EC/DEC-based electrolyte (**a**) and the diglyme-based electrolyte (**b**). **c** Separation (potential polarization) between the cathodic and anodic peaks in the CV curves as a function of scan rate. **d** *b*-values (slopes) obtained from the current peaks, *I*_p_ = *av*^*b*^, of the CV curves

To reveal the true nature of the polarization at the TiO_2_ working electrode, we designed a coin cell-type 3-electrode setup (Supplementary Figure 12) and performed scan rate-dependent CV measurements, by which the effect at the Na counter electrode was eliminated. The CV results obtained from these tests are quite similar to those observed in the 2-electrode measurements (Supplementary Figure 11), as shown in Fig. 3, except for the smaller polarizations. It can be seen from Fig. 3c that the TiO_2_ working electrode coupled with the EC/DEC-based electrolyte has a much higher polarization than when coupled with the diglyme-based electrolyte, and the polarization becomes more severe as the scan rate increases. The potential polarization with an increased scan rate is extremely small in the case of diglyme, indicating quite favorable sodiation dynamics of the TiO_2_ electrode. Additionally, CV shows that the sodium storage of TiO_2_ is controlled by both capacitance and diffusion, and using the diglyme-based electrolyte increases the contribution from capacitance and facilitates the rapid (de)intercalation of sodium ions. This is very similar to that observed in graphene-modified TiO_2_ where the polarization decreases^43^ and the Na ion intercalation pseudocapacitive process increases^24^.

The total charge stored in a CV scan can be separated into faradaic contributions from Na-ion intercalation and pseudo-capacitance, and a non-faradaic contribution from the double layer effect that depends on the surface area^44^. These capacitive effects (pseudo-capacitance and double layer charging) can be distinguished from Na-ion intercalation by analyzing the CV data at different sweep rates according to the relationship between the measured current (*I*) and the scan rate (*v*)^45^:

$$I = av^b$$

and the *b*-value is determined by the slope of the log(*v*)–log(*I*) plot. In particular, a *b*-value of 0.5 indicates total diffusion-controlled behavior, whereas 1.0 indicates a capacitive process. The log(*v*)–log(*I*_p_) plots and corresponding *b*-values are shown in Fig. 3d (note that here we use the peak current *I*_p_ rather than the current *I* at a given potential because of the large polarization), showing that in both cases the (de)sodiation is a combination of capacitance and diffusion. However, the *b*-values in the EC/DEC case are smaller than in the case of diglyme, meaning that for the EC/DEC the (de)sodiation is more diffusion-controlled and has slower diffusion kinetics than with diglyme. When using diglyme, a higher contribution of capacitive charge causes an increased intercalation pseudocapacitive behavior of Na^+^ in the electrode (considering the double layer charging is sensitive to the surface area and thus is the same no matter the electrolyte), which contributes to the excellent rate capability and high capacity^24^.

**Fig. 4:**
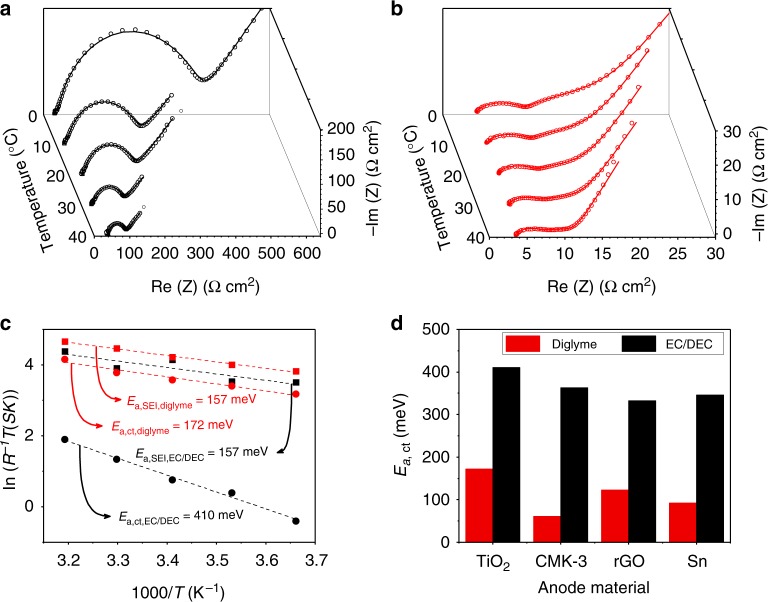
Electrochemical impedance spectroscopy analysis after ten discharge–charge cycles. **a**, **b** Temperature-dependent Nyquist plots of the TiO_2_ anode for the EC/DEC-based electrolyte (**a**) and the diglyme-based electrolyte (**b**). **c** Arrhenius plot of the resistance contributions of the solid electrolyte interphase *R*_SEI_ and the charge transfer resistance *R*_ct_, with the derived activation energies *E*_a,SEI_ and *E*_a,ct_ for the TiO_2_ anode in the two electrolytes. **d** Comparison of the charge transfer energy barriers in the diglyme-based electrolyte and EC/DEC-based electrolytes for various electrode materials

The increased intercalation pseudo-capacitance triggered by the diglyme-based electrolyte indicates faster sodium ion diffusion and electron transport across the whole cell. To gain more fundamental insight into the faster dynamics, we conducted temperature-dependent EIS to examine the dynamic properties (the resistance and activation energy *E*_a_) of individual components in the cell system. According to Barsoukov’s model^46^, the sodium ions diffuse across the liquid electrolyte (LE) and SEI layer, followed by a charge transfer process at the SEI/electrode interface and diffusion inside the electrode material. Here, we assume an SEI/electrode interface extending from the SEI layer to the electrode (as illustrated in Supplementary Figure 14). Different resistances and activation energies are involved. The resistances can be determined by fitting the EIS spectra, and the activation energies can be determined by fitting the temperature-dependent resistances. Figure 4a, b, respectively, shows the temperature-dependent EIS spectra of 2-electrode cells assembled using EC/DEC-based and diglyme-based electrolytes after 10 discharge–charge cycles at a rate of 100 mA g^−1^. Two semicircles and a straight line are identified in all these spectra, and in order to assign them, we compared the EIS spectra measured using both 2-electrode and 3-electrode setups (Supplementary Figure 13). It was found that the small semicircle at higher frequencies observed in the 2-electrode study almost disappears in the 3-electrode study, which implies that the first semicircle at higher frequencies in the 2-electrode study includes the information of the SEI at the Na counter electrode. The second semicircle at lower frequencies in the 2-electrode study is very similar to the semicircle observed in the 3-electrode study. The DC-bias current-dependent EIS study using the 3-electrode setup confirms that this semicircle may be assigned to the charge-transfer process (Supplementary Figure 13a). Therefore, in the following discussion, we use the 2-electrode EIS study. The Arrhenius plots in Fig. 4c and data in Supplementary Table 2 represent the individual contributions of the solid electrolyte interphase *R*_SEI_ and the charge transfer resistance *R*_ct_, obtained from data fitting the temperature-dependent impedance spectra based on the equivalent circuit given in Supplementary Figure 14. For both the SEI and the SEI/electrode interface, an Arrhenius equation is used to determine the activation energies^47^:

$$\sigma T = A{{\rm exp}}\left( { - E_{\rm a}/k_{\rm B}T} \right).$$

Here, *A* is the pre-exponential factor, *E*_a_ is the apparent activation energy for ion transport, *k*_B_ is the Boltzmann constant, *σ* is the ionic conductivity, and *T* is the absolute temperature. As expected, transport across the SEI/electrode interface has higher activation energy (*E*_a*,* ct_) than that across the SEI layer (*E*_a*,* SEI_) for both cases. When the EC/DEC-based electrolyte is used, the activation energy across the interface (*E*_a,ct, EC/DEC_) is the highest with a value of ~410 meV (which is very close to the value of ~400 meV from the 3-electrode EIS study, as shown in Supplementary Figure 13b, c and Supplementary Table 1, further verifying the effectiveness of the analysis in the 2-electrode study), and is higher by a factor of 2.4 than the corresponding value for the diglyme-based electrolyte. This means that at any temperature, transport of Na ions is faster across the SEI/electrode interface formed in diglyme than that formed in EC/DEC. The activation energy of Na^+^ diffusion in the SEI layer *E*_a, SEI_ is almost the same for the two cases. This is probably due to the fact that Na^+^ diffuses in the SEI layer by hopping migration and the activation energy for this is not significantly different in different SEI compositions^48,49^. It is also worth noting that the interfacial ionic conductivity in the diglyme-based system is much higher than that in the EC/DEC-based system, which implies a major effect of the use of different solvent-based electrolytes in SIBs. If transport through the SEI/electrode interface is sluggish, this interfacial resistance will have a detrimental effect on the rate capability^47^. This finding provides crucial insight into the improved interfacial characteristics induced by ether-based electrolytes, which is also demonstrated for other electrode materials, as shown in Fig. 4d. We also examined the temperature-dependent EIS of a CMK-3 anode (Supplementary Figure 15 and Table 3), an rGO anode (Supplementary Figure 16 and Table 4), and an Sn anode (Supplementary Figure 17 and Table 5) after cycling. In all cases, the charge transfer energy barrier (*E*_a, ct_) across the interface in the EC/DEC-based electrolyte is much higher than that in the diglyme-based electrolyte, by a factor of around 6 for a CMK-3 anode, 2.7 for an rGO anode, and 4 for an Sn anode.

### SEI analysis

**Fig. 5:**
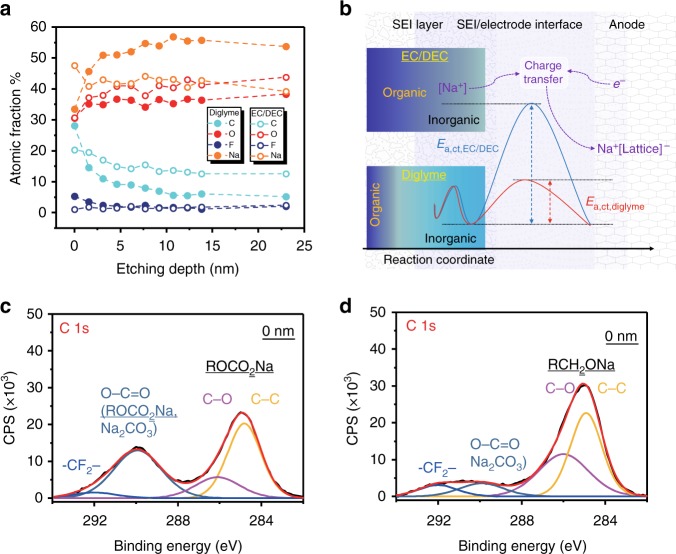
SEI chemistry and the charge transfer process. **a** The atomic fractions of C, O, F, and Na as a function of SEI depth, calculated from the XPS spectra depth profiling (Supplementary Figure 19). **b** Schematic of the SEI compositions formed in different electrolytes and a comparison of the charge transfer energy barriers. [Na^+^] means that the Na^+^ may be solvated. There is a smaller amount of organic species in the SEI formed in the diglyme-based electrolyte and the inner part of the SEI is mainly composed of inorganic species. **c** XPS C 1s spectrum before etching in the EC/DEC-based electrolyte. **d** XPS C 1s spectrum before etching in the diglyme-based electrolyte

SEM examination of the electrode surface showed that an SEI had formed after cycling (Supplementary Figure 18). It seems that the formation of the SEI in a diglyme-based electrolyte is more uniform than that formed in an EC/DEC-based electrolyte. We therefore carried out XPS to examine the SEIs formed in the two electrolytes, as shown in Fig. 5a–d. XPS depth profiling was done using Ar ion sputtering. As shown in Supplementary Figure 19, C, O, F, and Na signals were detected on the electrode surface in both cases and also at different etching depths. Figure 5a shows the elemental compositions of the SEI layer at different depths obtained by quantitative analysis of the XPS spectra. The most significant feature is that in the SEI formed in the diglyme-based electrolyte, the atomic fraction of C decreases distinctly with increasing etching depth (~28% at the surface and ~5% at 23 nm depth), while the atomic fraction of Na shows a completely opposite trend, and the atomic fraction of O increases slightly with etching depth. This means that the organic species are mainly distributed near the surface while the interior of the SEI is mainly composed of inorganic species. Although a slight decrease in the atomic fraction of C in the SEI formed in the EC/DEC-based electrolyte with etching depth is also observed, its value is much higher than with the diglyme counterpart. This indicates that there are more organic species in the SEI formed in the EC/DEC-based electrolyte and they are spread over the whole thickness of the SEI. Analyses of the Na Auger Parameter confirms these conclusions (Supplementary Figure 21 and Supplementary Table 6)^50^. The inorganic species are generally localized and remain in a stable thin layer during cycling, leading to a low charge transfer resistance and energy barrier^16,49^. The presence of organic products, which are usually porous, makes the SEI much thicker^16^, leading to a much higher charge transfer resistance and energy barrier.

The SEI composition was also analyzed by high-resolution XPS using a Mg X-ray source. First, we analyzed the composition of the SEI formed in the diglyme-based electrolyte (Fig. 5d, Supplementary Figures 22–26 and Supplementary Table 7). The C 1s spectrum was fitted using four peaks with binding energies of ~284.9 eV (C–C), ~286.0 eV (C–O), ~289.9 eV (O–C=O), and ~292.0 eV (–CF_2_–), which are consistent with sodium alkoxides (RCH_2_ONa) being the main reduction products of diglyme and Na_2_CO_3_ and PVDF^9,16,50^. The O 1s spectrum contained three peaks at ~532.6 eV (R–O–Na), ~531.1 eV, and ~530.0 eV (Na–O–Ti), belonging to RCH_2_ONa, Na_2_CO_3_, and Na_*x*_TiO_2_, respectively. The O 1s spectrum change with etching depth also proves that the organic species decrease in the inner region of the SEI (Supplementary Figure 24a–c). The F 1s spectrum showed a peak at ~684.9 eV (Na–F) and the Na 1s spectrum showed a peak at ~1072.2 eV (Na–F, Na–O), indicating the existence of NaF and other sodium compounds (RCH_2_ONa, Na_2_CO_3_). Overall, we concluded that the SEI formed in the diglyme-based electrolyte contains both organic (RCH_2_ONa) and inorganic (Na_2_CO_3_, NaF) components. In the case of the EC/DEC-based electrolyte, we saw distinct XPS peaks corresponding to sodium alkyl carbonate (ROCO_2_Na) and inorganic products (Na_2_CO_3_, NaF), as shown in Fig. 5c, Supplementary Figures 22–26, and Supplementary Table 8 (refs. ^9,16,50–54^). Therefore, in addition to the distinct composition change along the SEI depth, there is also a difference between the SEIs formed in the two electrolytes because of the organic components formed by the decomposition of the different solvents, which is consistent with what is found in SIB Bi electrodes^15^. It has been reported that sodium alkoxides (RCH_2_ONa) ensure fast Na^+^ transport and are essential for the interfacial stability of the Bi electrode^15^. First-principles calculations conducted by Su et al. demonstrated that an ether-based solvent reduces the energy barrier for sodium-ion diffusion and thus improves the electrochemical performances^11^. It is reasonable to deduce that the RCH_2_ONa also reduces the energy barrier for sodium-ion diffusion. The inorganic species in the SEI generally have low Na^+^ diffusion activation energies (e.g., the activation energy for NaF is ~100–150 meV^49^). Combined with the above dynamic analysis, we conclude that the different SEI chemistries lead to the huge difference in the electrochemical performance of the two different solvent-based electrolytes by modifying the resistance of the SEI/electrode interfaces and the transport of Na^+^ and electrons in them.

## Discussion

The dynamic properties of the SEI/electrode interface play a vital role in the sodium storage. Modification of this interface can be achieved by changing the SEI chemistry, which depends on whether an ether-based or an ester-based electrolyte is used. But how the SEI chemistry works behind-the-scenes has not yet been fully determined, and undoubtedly needs further in situ experimental investigation and theoretical analysis. To support the governing role of the SEI/electrode interface, we measured the electrochemical performance and analyzed the sodiation dynamics of an anatase TiO_2_ anode composed of 84 nm nanocrystals (Supplementary Figure 26). First, as shown in Supplementary Figure 27, this anode shows similar voltage profiles to those of the 16 nm TiO_2_ nanocrystal anode but has relatively lower specific capacities in both the EC/DEC-based and diglyme-based electrolytes, with the capacity in the latter being higher than in the former, similar to what is observed for the 16 nm nanocrystal anode. We hypothesize that the reasons for the inferior capacity of 84 nm nanocrystal material are the high sodium diffusion energy barrier and relatively small crystal surface area. Interestingly, dynamic analysis shows that in the diglyme-based electrolyte, as shown in Supplementary Figure 28, 29 and Table 9, the activation energies of sodium diffusion through the SEI layer as well as the SEI/electrode interface are quite close for the two materials, meaning almost the same energy barriers for sodium transport and charge transfer. On the one hand, this indicates that the activation energy for charge transfer at the electrolyte/electrode interface is inherent to the nature of the interface and is almost independent of particle size. On the other hand, the large surface area is one of the reasons accounting for the better performance of the 16 nm than the 84 nm material, because a larger surface area results in a correspondingly larger interface area and higher electrochemical reactivity.

Apart from the electrolyte/electrode interfacial properties, solvent wettability is another parameter that may influence the sodium ion surface diffusion kinetics. Better solvent wettability is beneficial for improving the rate performance because of better ion availability as a result of the easier penetration of the electrolyte, since the surface ion availability may play an essential role in providing sufficient Na ions for intercalation into the TiO_2_^55^. The solvent wettability is measured by the contact angle. As shown in Supplementary Figure 30, both the EC/DEC-based and diglyme-based electrolytes effectively spread over the electrode surface composed of 16 nm TiO_2_ nanocrystals, super P and PVDF within 5 s, suggesting that the electrolyte wettability is not a decisive factor that influences the battery performance of the 16 nm TiO_2_ anode. While for the electrode consisting 84 nm nanocrystals, the wettability is distinctly different for the two electrolytes (Supplementary Figure 30) with that of the diglyme-based electrolyte being better than that of the EC/DEC-based electrolyte. Except for the larger electrochemical active surface area, the better electrolyte wettability should also contribute to the superior electrochemical performance of the 16 nm TiO_2_ compared to the 84 nm TiO_2_.

In summary, as a superior model anode, the 16 nm nanocrystal anatase TiO_2_ coupled with the diglyme-based electrolyte shows a reversible capacity of 257.9 mAh g^−1^ at 100 mA g^−1^ and more than 100 mAh g^−1^ at 2000 mA g^−1^, both of which are much better than in the EC/DEC-based electrolyte and are among the best values ever reported for anatase-based anodes for SIBs. The Sn, rGO, and CMK-3 anodes also show superior Na^+^ storage performance in the diglyme-based electrolyte than in the EC/DEC-based electrolyte. In operando Raman spectra and XRD show that the ether-based electrolyte facilitates the sodiation-induced structural transitions of the TiO_2_ and Sn anodes. We have also shown that charge transfer dynamics at the SEI/electrode interface play a crucial role in the rate-cycling performance and are nearly size-independent. Charge transfer has an energy barrier of 172 meV for the interface formed in the diglyme-based electrolyte (1.4 times lower than for the interface in the EC/DEC-based electrolyte) for the TiO_2_ anode, providing faster charge transfer across the electrolyte/electrode interface. Significant reductions in the charge transfer energy barrier are also demonstrated in Sn, CMK-3, and rGO electrodes when the EC/DEC-based electrolyte is replaced by the diglyme-based electrolyte. The faster dynamics increase the Na^+^ intercalation pseudocapacitive behavior, which is highly beneficial to fast charge storage and long-term stability. The chemistry and composition distribution in the SEI layer are of great relevance for the charge transfer, with the organic species formed in the ether-based electrolyte together with the inorganic species favoring the sodiation dynamics. The information obtained in this work provides a general framework for understanding the origin of the extraordinary performance and improved interfacial characteristics of an ether-based electrolyte for SIB anodes, and can guide the future design and matching between non-aqueous electrolytes and novel electrode materials for rechargeable batteries.

## Methods

### Synthesis of anatase TiO_2_ nanocrystals

Titanium(IV) butoxide (TB, 97%), oleic acid (OA, 90%), oleylamine (OM, 70%), and absolute ethanol were purchased form Aldrich. All chemicals were used as received. The synthesis of the TiO_2_ nanocrystals was accomplished using a solvothermal method. Typically, 5 mmol TB was added to a mixture of 25 mmol OA, 25 mmol OM, and 100 mmol absolute ethanol. The mixture was stirred in a 40-mL Teflon cup for 10 min before being transferred into a 100-mL Teflon-lined stainless-steel autoclave containing 20 mL of a mixture of ethanol and water (96% ethanol, v/v). The system was then heated at 180 °C for 18 h. The white TiO_2_ nanocrystal precipitates obtained were washed several times with ethanol and then dried at room temperature before being heated at 350 °C for 5 h in air.

### Materials characterization

Transmission electron microscope (TEM) images and selected area electron diffraction (SAED) patterns of the TiO_2_ nanocrystals were obtained using a JEOL 2010F transmission electron microscope operated at 200 kV. Raman spectra were collected using a Raman spectrometer (LabRAM HR spectrometer, Horiba) with a 532.05-nm Ar-ion laser. XRD patterns were recorded over the range 20–80° on a Rigaku diffractometer equipped with a Cu K_α_ radiation source operated at 40 kV and 120 mA.

### Cell assembly and testing

The active materials (70 wt%) were homogeneously mixed with conductive carbon black (15 wt%) and PVDF (15 wt%) in NMP to form a slurry, which was stirred for 6 h and coated onto a Cu foil. After vacuum-drying at 110 °C for 10 h, the electrodes were cut into circular pieces with a diameter of 12 mm, which were used as anodes for the assembled cell. The mass loading of the electrodes was 1.5 mg cm^−2^. The electrolytes for comparison were 1 M NaCF_3_SO_3_ dissolved in a mixture of EC and DEC (volume ratio = 1:1), and 1 M NaCF_3_SO_3_ dissolved in diglyme. Using these electrodes and electrolytes with pure sodium foils as counter electrodes and Whatman GF/D glass fibers as separators, 2032-type coin cells were assembled in an argon-filled glove box with oxygen and moisture contents below 0.1 ppm. Galvanostatic charge–discharge tests were conducted on a LAND CT2001 battery program controlling system at different current densities with a voltage window of 0.005–2.5 V vs. Na/Na^+^.

### Cyclic voltammetry

CV measurements were conducted using an electrochemical workstation (VMP3, Bio Logic, France) at scanning rates from 0.1 mV s^−1^ to 10 mV s^−1^ in the voltage range of 0.005–2.5 V vs. Na/Na^+^ at room temperature.

### Electrochemical Impedance Spectroscopy

All EIS measurements were carried out using a PARSTAT 4000 electrochemical workstation, using a frequency range of 10^5^ Hz to 100 mHz and an AC voltage amplitude of 10 mV. The cell temperature was controlled in a temperature-controlled chamber (Linkam Scientific) and was varied between 0 and 40 °C (±0.1 °C) in steps of 10 °C using a nitrogen-gas flow and electronic heaters (Linkam Scientific) for the temperature-dependent experiments.

### In operando Raman spectroscopy and in situ XRD

Raman spectra acquisition during cell operation has been described in detail elsewhere and is schematically shown in Supplementary Figure 5 (ref. ^39^). For the measurements, a special electrode was used which was prepared by mixing the active materials with super P and 5 wt% PTFE in water to obtain a homogenous slurry. The final content of the active materials was 80 wt%. The slurry was rolled into a thin film, which was then pressed onto a stainless-steel mesh. A delicate battery cell with a quartz window on the top was used (Supplementary Figure 5). The Raman spectra were recorded on a MicroRaman system (LabRAM HR spectrometer, Horiba) with an Olympus BX microscope and an argon ion laser (532.05 nm). Each spectrum was acquired for 20 s. The galvanostatic discharge of the cell was controlled by an electrochemical workstation (PARSTAT 4000). A similar battery cell to that used for the in operando Raman spectroscopy was designed, replacing the quartz window with a Kapton film window to perform the in situ XRD measurements (Supplementary Figure 8).

### Surface characterization

XPS survey scan analyses were conducted on a PHI 5000 VersaProbe II spectrometer using a monochromatic Al K_α_ X-ray (1486.6 eV) source. High-resolution XPS analyses were carried out using an ESCALAB 250Xi spectrometer with a Mg K_α_ X-rays source, in order to eliminate the interferences of the Na Auger peak with the oxygen 1s photoelectron line and the Ti Auger peak with the Na 1s peak. The batteries were disassembled in a glove box after 10 discharge–charge cycles at a rate of 100 mA g^−1^, and the anodes were washed several times with the related solvent, i.e., dimethyl carbonate (DMC) for EC/DEC and dimethoxyethane (DME) for diglyme. After drying, the anodes were transferred to a vacuum box and then transferred into the XPS chamber. Samples were ion-etched using 2 kV Ar ions over 2 × 3 mm^2^ with an etching rate of approximately 4.3 nm min^−1^. Spectra were charge corrected to the main line of the carbon 1s spectrum (adventitious carbon) and set to a BE of 284.8 eV (Supplementary Figure 21).

## Supplementary information

Supplementary Information

## Data Availability

The data that support the findings of this study are available within the article and its Supplementary Information files. All other relevant data supporting the findings of this study are available from the corresponding authors upon reasonable request.
