# Author Correction: Evolution of the electrochemical interface in sodium ion batteries with ether electrolytes

**DOI:** 10.1038/s41467-019-09129-6

**Published:** 2019-03-13

**Authors:** Kaikai Li, Jun Zhang, Dongmei Lin, Da-Wei Wang, Baohua Li, Wei Lv, Sheng Sun, Yan-Bing He, Feiyu Kang, Quan-Hong Yang, Limin Zhou, Tong-Yi Zhang

**Affiliations:** 1 Shenzhen Environmental Science and New Energy Technology Engineering Laboratory, Tsinghua-Berkeley Shenzhen Institute (TBSI), Tsinghua University, Shenzhen, 518055 China; 2 Interdisciplinary Division of Aeronautical and Aviation Engineering, The Hong Kong Polytechnic University, Hong Kong, China; 3 Department of Mechanical Engineering, The Hong Kong Polytechnic University, Hong Kong, China; 4 School of Chemical Engineering, The University of New South Wales, Sydney, 2052 NSW Australia; 5 Shenzhen Key Laboratory for Graphene-based materials and Engineering Laboratory for Functionalized Carbon Materials, Graduate School at Shenzhen, Tsinghua University, Shenzhen, 518055 China; 6 Materials Genome Institute, Shanghai University, 333 Nanchen Road, 200444 Shanghai, China; 7 Nanoyang Group, State Key Laboratory of Chemical Engineering, School of Chemical Engineering and Technology, Tianjin University, 300072 Tianjin, China

Correction to: *Nature Communications*; 10.1038/s41467-019-08506-5; published online 13 February 2019

The original version of this Article incorrectly omitted an affiliation of Kaikai Li: ‘Shenzhen Environmental Science and New Energy Technology Engineering Laboratory, Tsinghua-Berkeley Shenzhen Institute (TBSI), Tsinghua University, Shenzhen 518055, China’. This has been corrected in both the PDF and HTML versions of the Article.

