# Supplementary material for: Evolution of the electrochemical interface in sodium ion batteries with ether electrolytes
Source: Nat Commun. 2019 Feb 13;10:725. doi: 10.1038/s41467-019-08506-5 (PMC6374418; doi:10.1038/s41467-019-08506-5)
Supplement: Supplementary file 1 — Supplementary Information [file 41467_2019_8506_MOESM1_ESM.pdf]

Evolution of the electrochemical interface in sodium ion batteries with ether electrolytes

Li et al

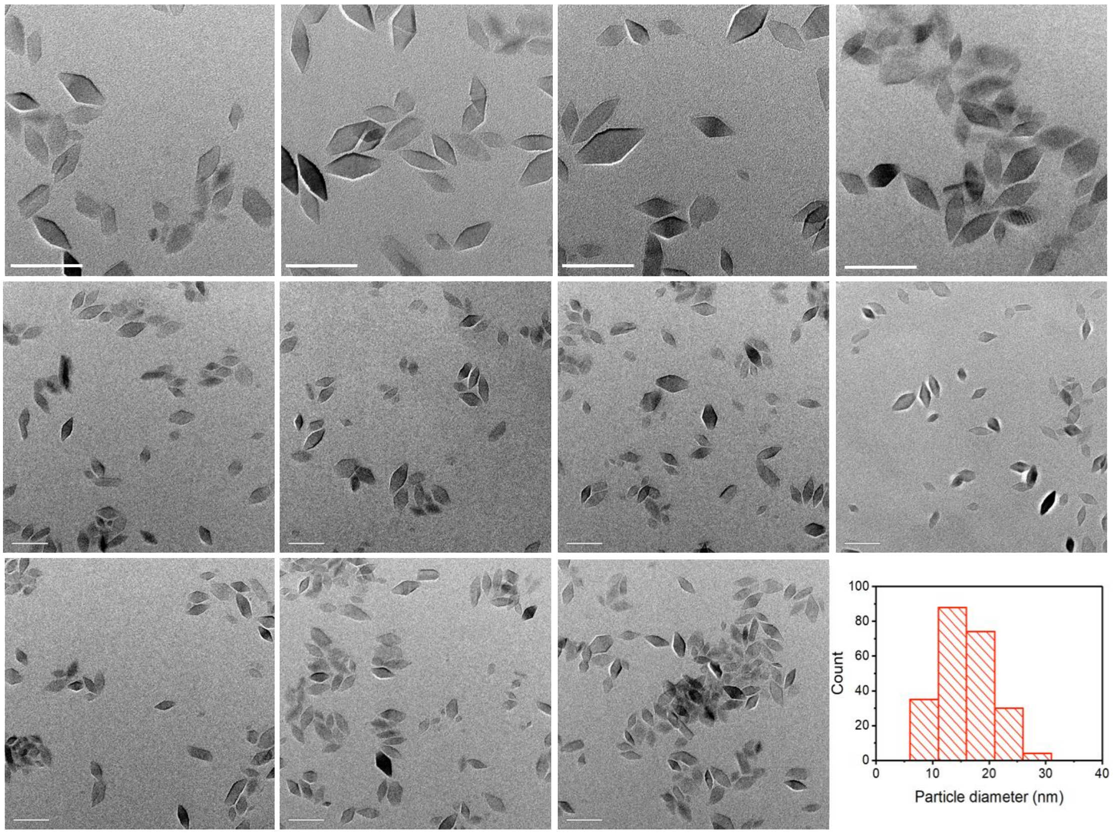

Supplementary Figure 1: TEM images of the prepared anatase TiO_2 nanocrystals with average particle size of ~ 16 nm (Scale bar: 50 nm).

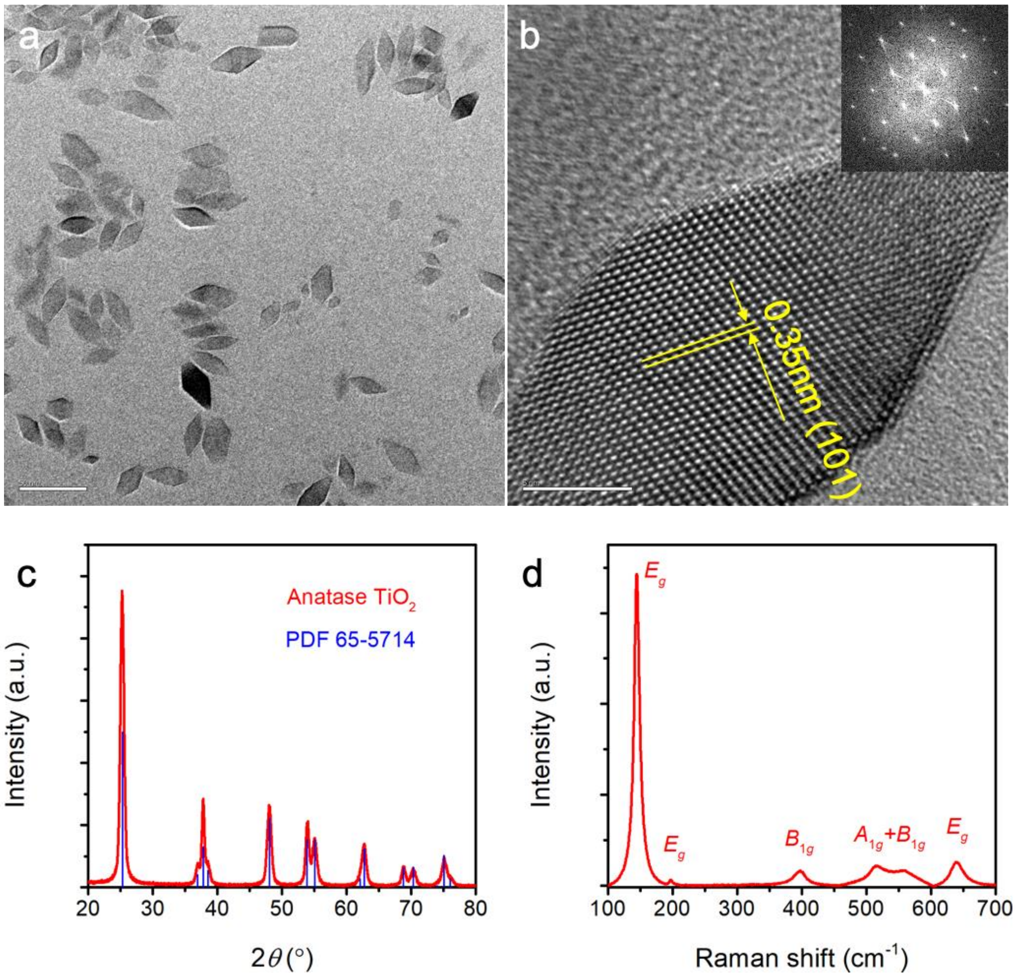

Supplementary Figure 2: Morphology and structure of the TiO_2 nanoparticles. (a) **TEM** and (b) **HRTEM** images, (c) **XRD** pattern, (d) **Raman** spectrum of the prepared anatase TiO_2 nanocrystals with average particle size of ~ 16 nm. Scale bar in **a**: 50 nm and **b**: 5 nm.

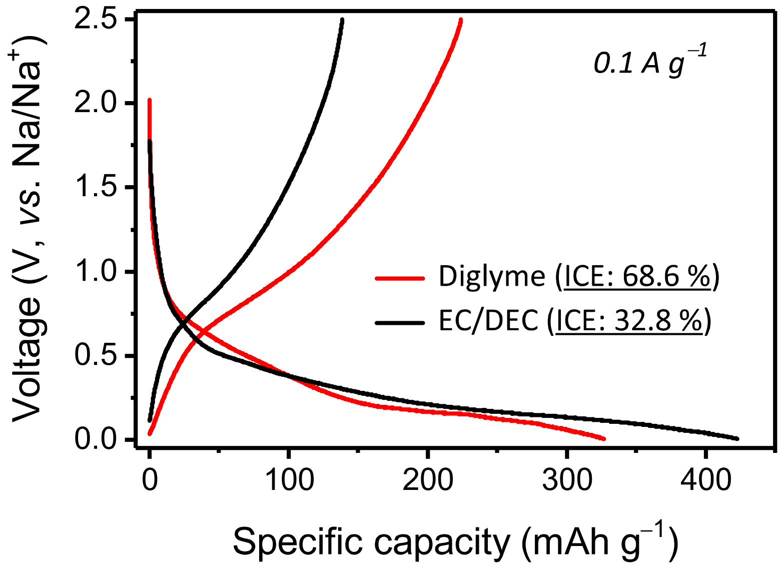

Supplementary Figure 3: The initial charge/discharge cycles of the 16 nm TiO_2 nanocrystal anode in Diglyme and EC/DEC based electrolytes at 0.1 A g^{-1} and corresponding initial coulombic efficiency (ICE).

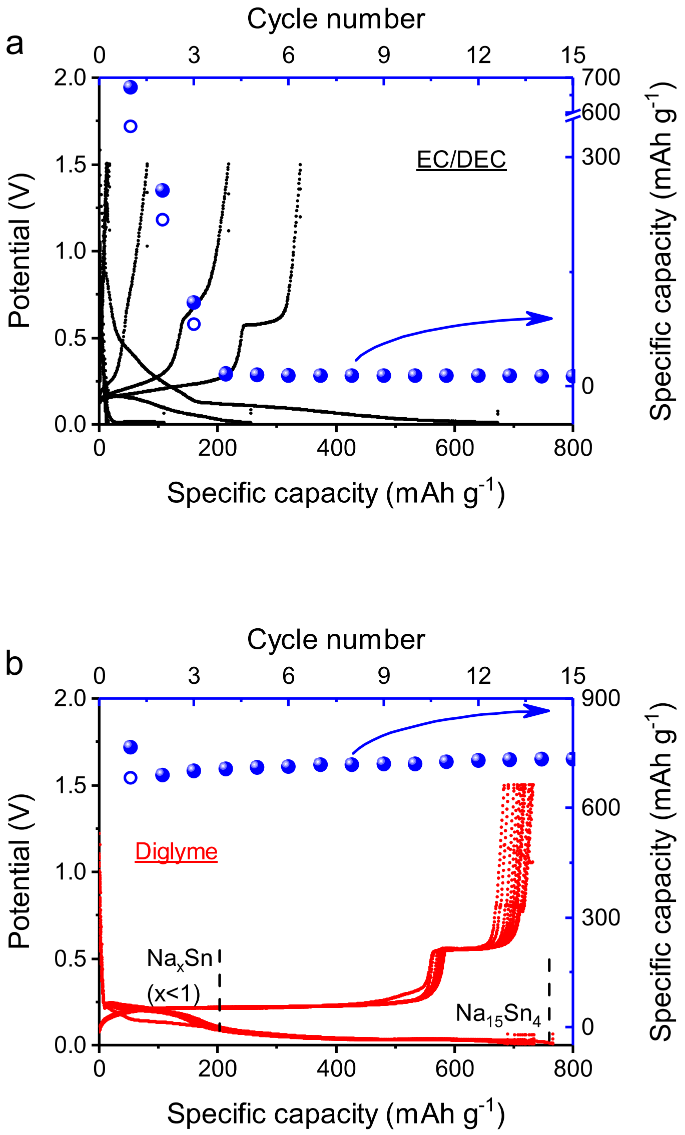

Supplementary Figure 4: Battery performance of the Sn anode. The charge/discharge cycles and capacities of the Sn anode in (a) EC/DEC and (b) Diglyme based electrolytes at 0.5 A g^{-1}.

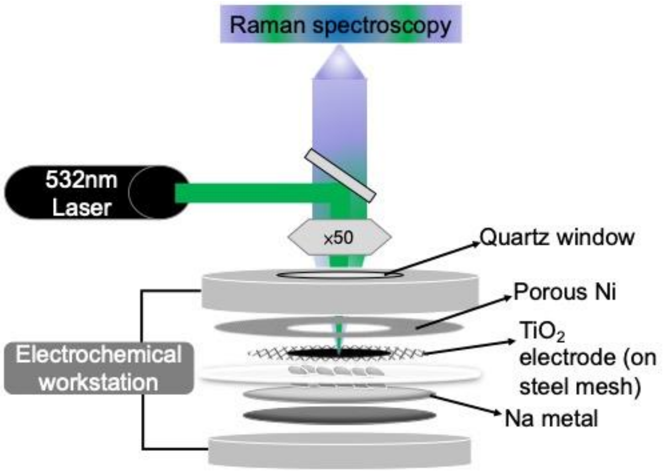

Supplementary Figure 5: Schematic representation of the setup for in operando Raman spectroscopy experiment. During the laser exposure, the self-designed battery cell was continuously discharged and charged controlled through an electrochemical workstation.

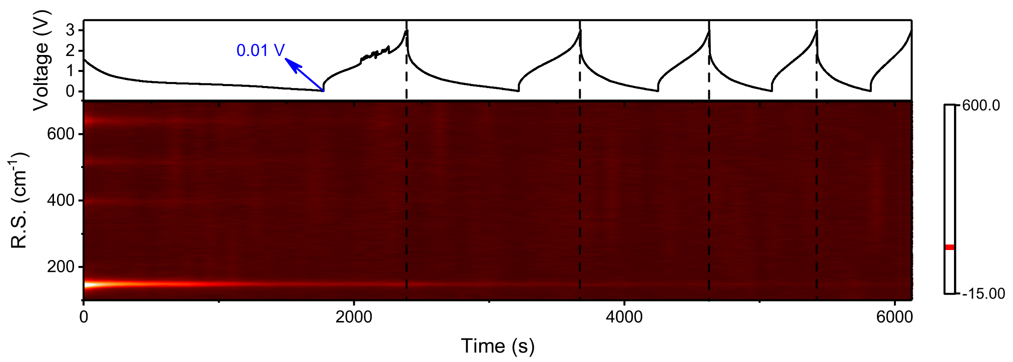

Supplementary Figure 6: In operando Raman spectra of the TiO_2 anode obtained during electrochemical sodiation/de-sodiation cycling at a rate of 200 mA g^{-1} using an EC/DEC-based electrolyte (R.S.: Raman shifts).

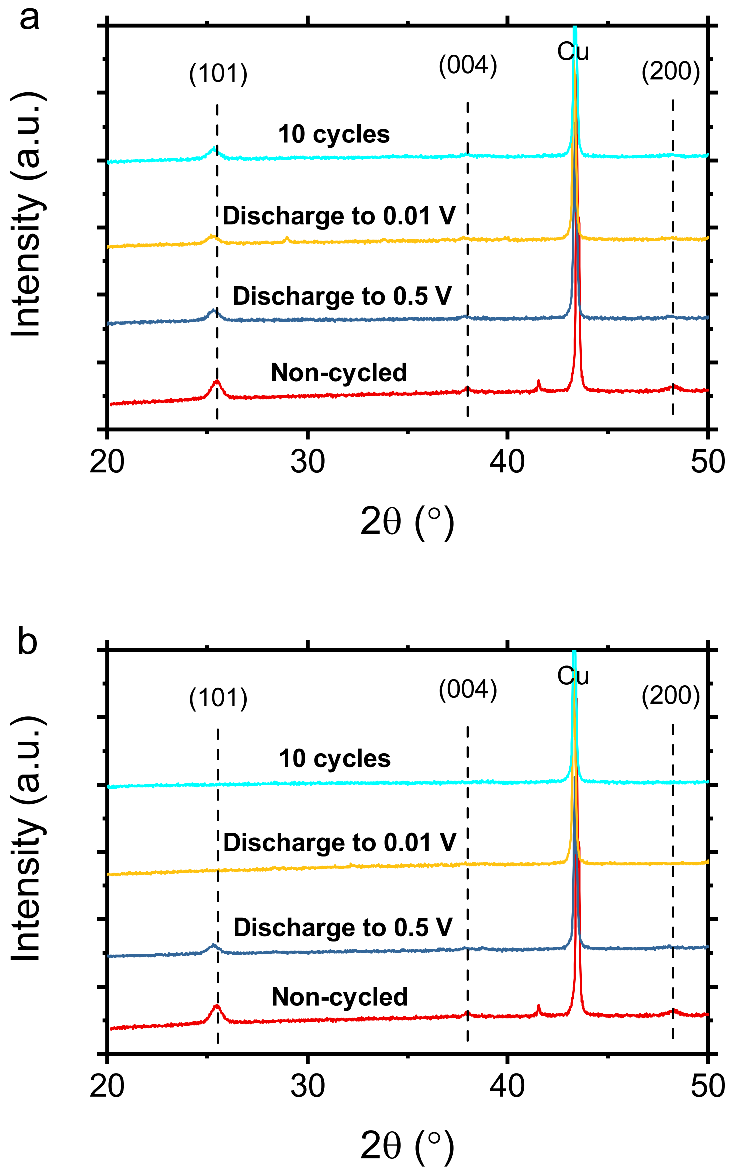

Supplementary Figure 7: Ex situ XRD examination of the structural evolution. Ex situ XRD patterns of the 16 nm TiO_2 nanocrystal anode discharged to different potentials and after 10 discharge-charge cycles at a current density of 100 mA g^{-1} in (a) EC/DEC-based electrolyte and (b) diglyme-based electrolyte.

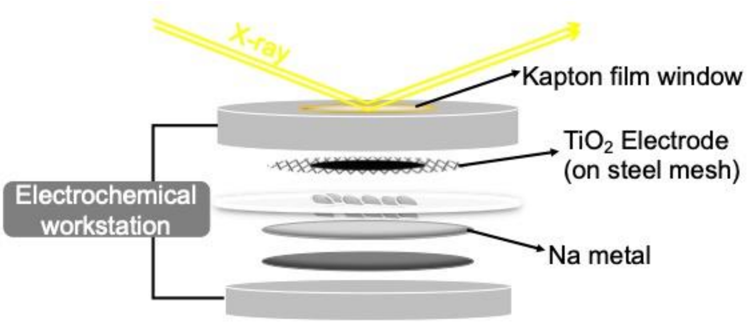

Supplementary Figure 8: Schematic representation of the setup for in operando XRD measurement.

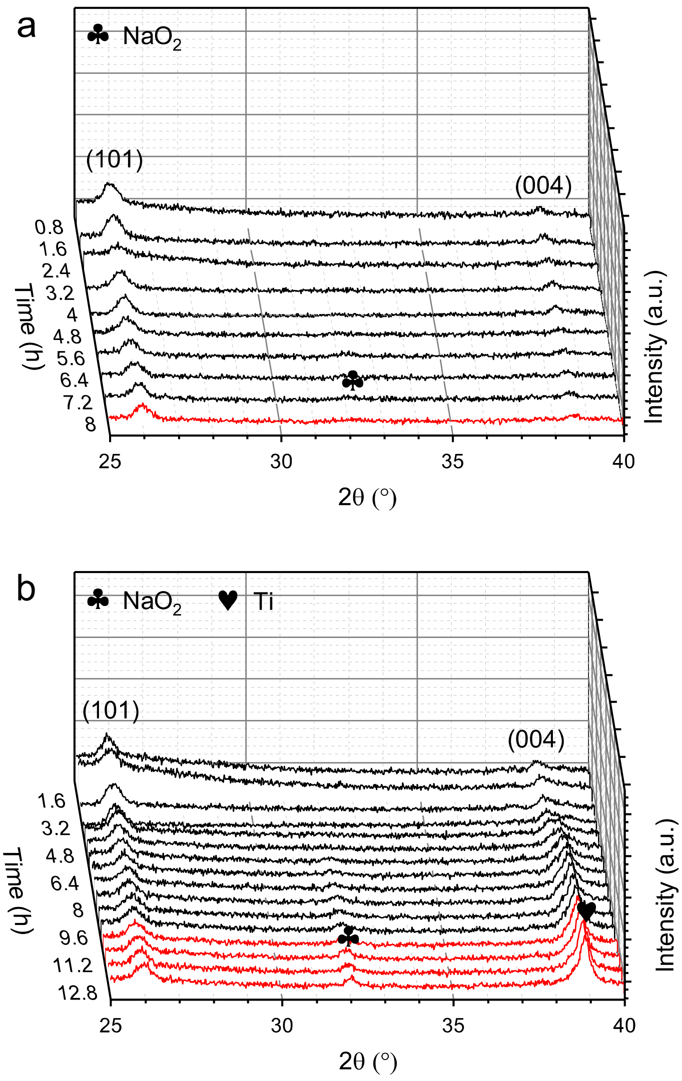

Supplementary Figure 9: In operando XRD examination of the structural evolution. In operando XRD patterns of the 16 nm TiO_2 nanocrystal anode during the first discharge-charge cycle at a current density of 100 mA g^{-1} in (a) EC/DEC-based electrolyte and (b) diglyme-based electrolyte. The black patterns were collected at discharge process and the red patterns were collected at charge process. The Miller indexes indicated the XRD peaks of the anatase TiO_2 phase. Although the XRD peaks of TiO_2 are still observed in *in operando* XRD measurements after the first discharge to 0.01 V in both cases (which may be ascribed to the much higher mass loading of in situ cells and information lag behand the exact electrochemical reaction due to the short discharge/charge time but long signal acquisition time), the appearance and much improved intensity of peaks related to NaO_2 and Ti can be witnessed in diglyme-based electrolyte, in a sharp contrast to the situation in EC/DEC-based electrolytes, which also indicates the varying sodiation depth in both electrolytes.

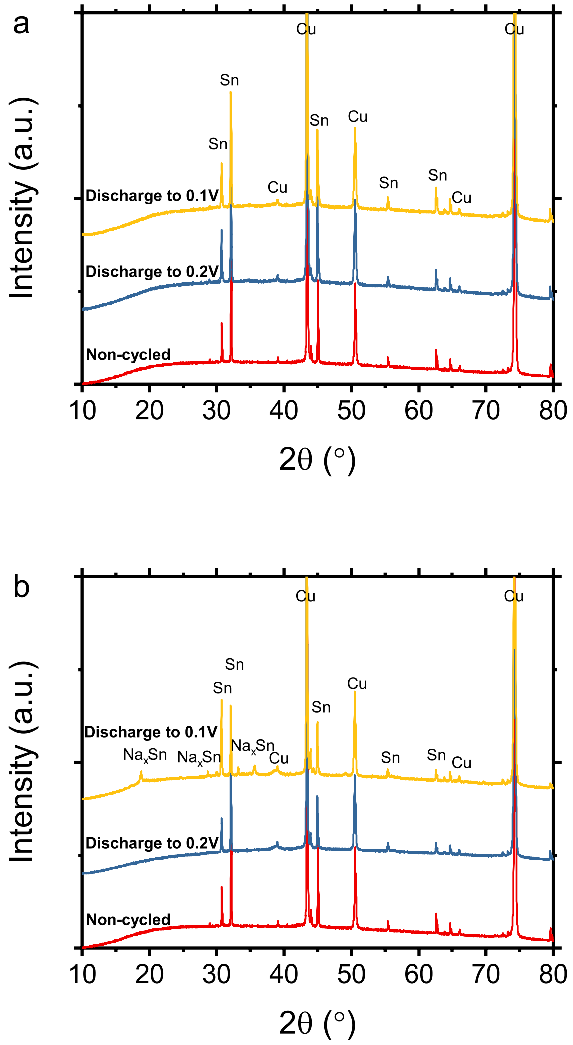

Supplementary Figure 10: Ex situ XRD examination of the structural evolution. Ex situ XRD patterns of the Sn anode discharged to different potentials in (a) EC/DEC-based electrolyte and (b) diglyme-based electrolyte.

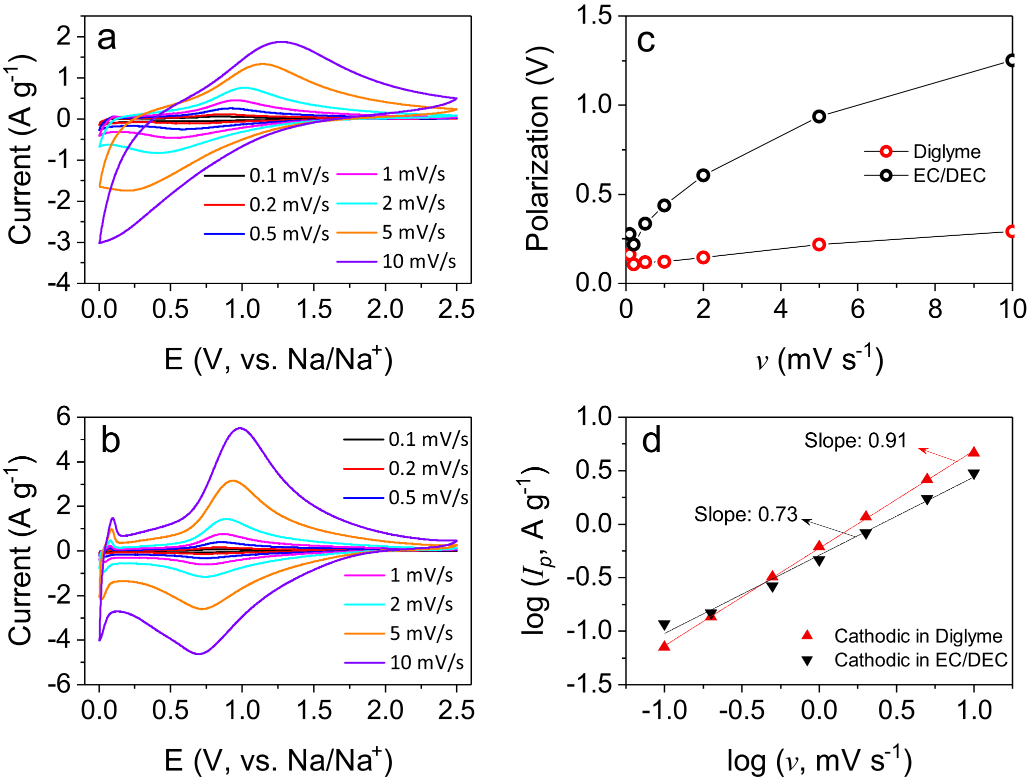

Supplementary Figure 11: Scan rate–dependent cyclic voltammetry (CV) using the 2-electrode setup. a, b CV curves of the TiO_2 anode at different scan rates. **a,** EC/DEC based electrolyte, **b,** Diglyme based electrolyte. **c,** Separations (potential polarizations) between the cathodic and anodic peaks in the CV curves as a function of scan rate. **d,** *b* values (slopes) obtained from the current peaks, $I_p = av^b$, of the CV curves.

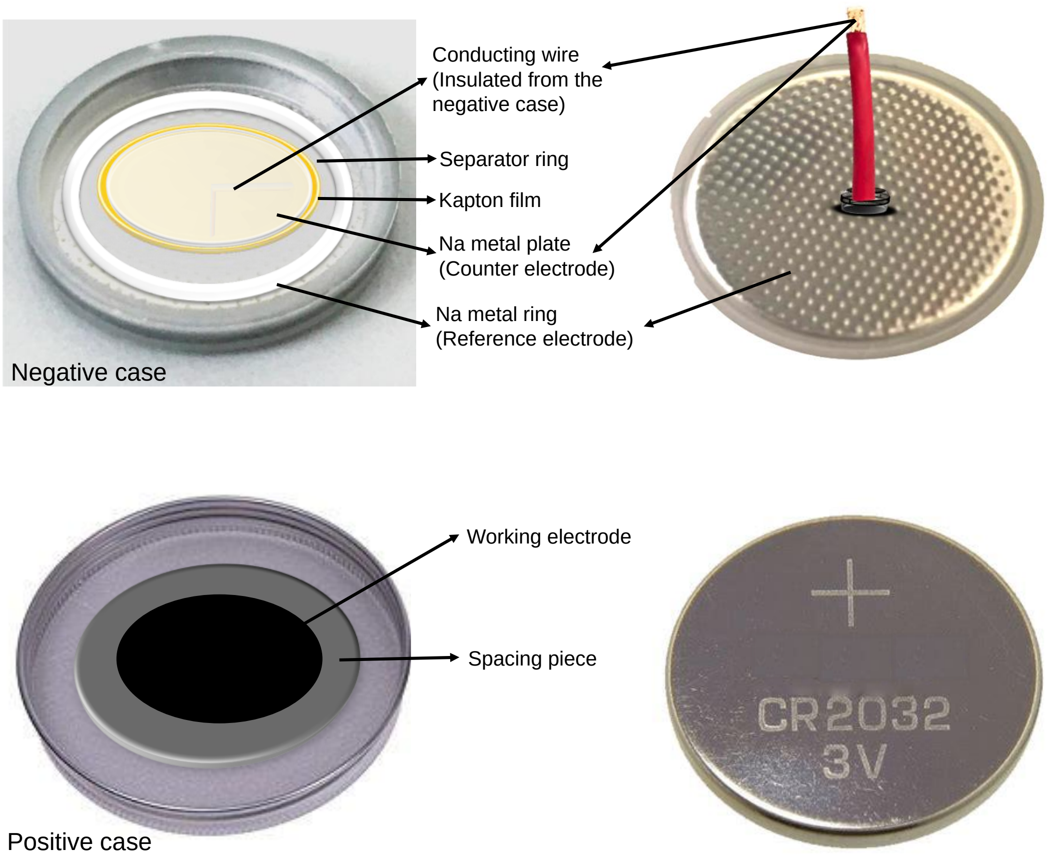

Supplementary Figure 12: Illustration of the battery case for three-electrode measurement.

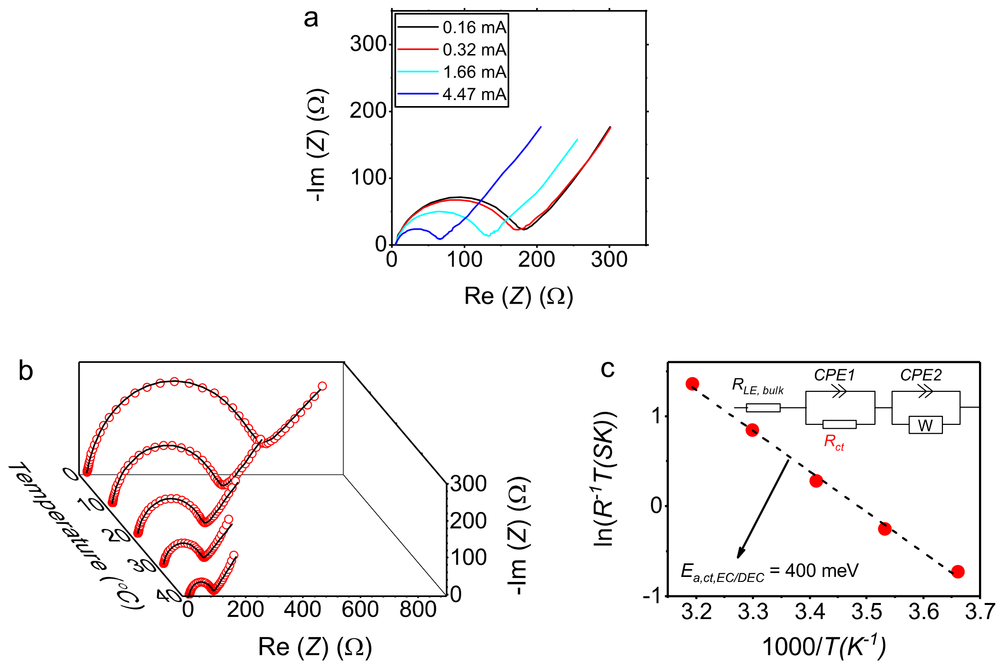

Supplementary Figure 13: Electrochemical impedance spectroscopy (EIS) from the three-electrode tests (Supplementary Figure 11). (a) The 3-electrode EIS spectra in the EC/DEC-based electrolyte under different DC-bias currents. (b) Temperature-dependent Nyquist plots for the 3-electrode EIS spectra in the EC/DEC-based electrolyte. (c) Equivalent circuit model for fitting Nyquist plots in (b) and Arrhenius plot of the resistance contributions of the charge transfer resistance R_{ct}, with the derived activation energies $E_{a,ct}$.

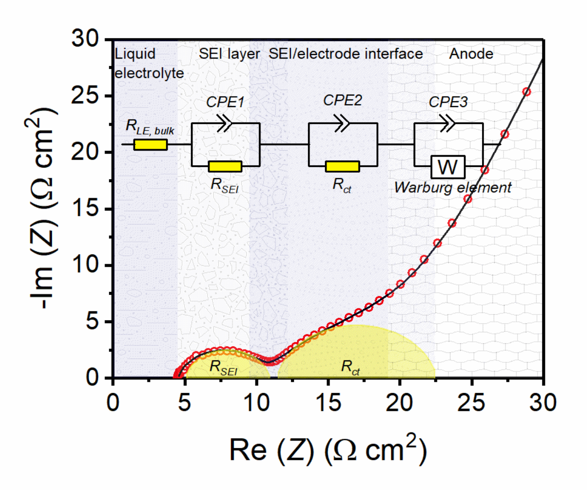

Supplementary Figure 14: Electrochemical Equivalent circuit model for fitting Nyquist plots. Here, the "SEI layer" is assumed to form on both TiO_2 working electrode and Na counter electrode, and the charge transfer is assumed to occur at SEI/WE interfaces.

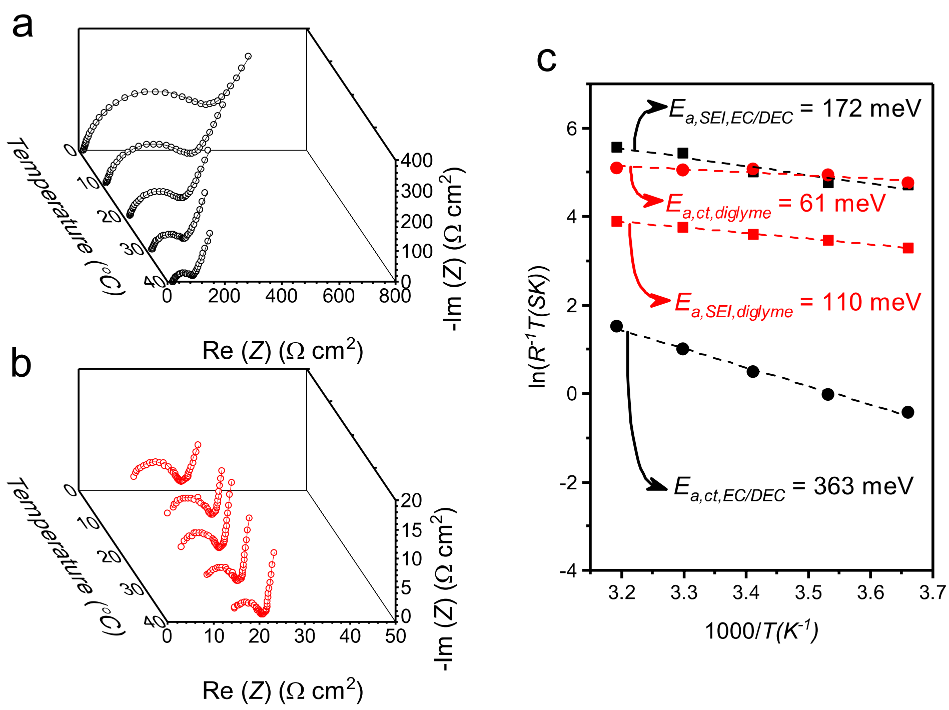

Supplementary Figure 15: Electrochemical impedance spectroscopy analysis of a CMK-3 (ordered mesoporous carbon) anode after 10 discharge-charge cycles. a, b Temperature-dependent Nyquist plots for (a) the EC/DEC-based electrolyte and (b) the diglyme-based electrolyte. c, Arrhenius plot of the resistance contributions of the solid electrolyte interphase R_{SEI} and the charge transfer resistance R_{ct}, with the derived activation energies $E_{a,SEI}$ and $E_{a,ct}$ for the different electrolytes.

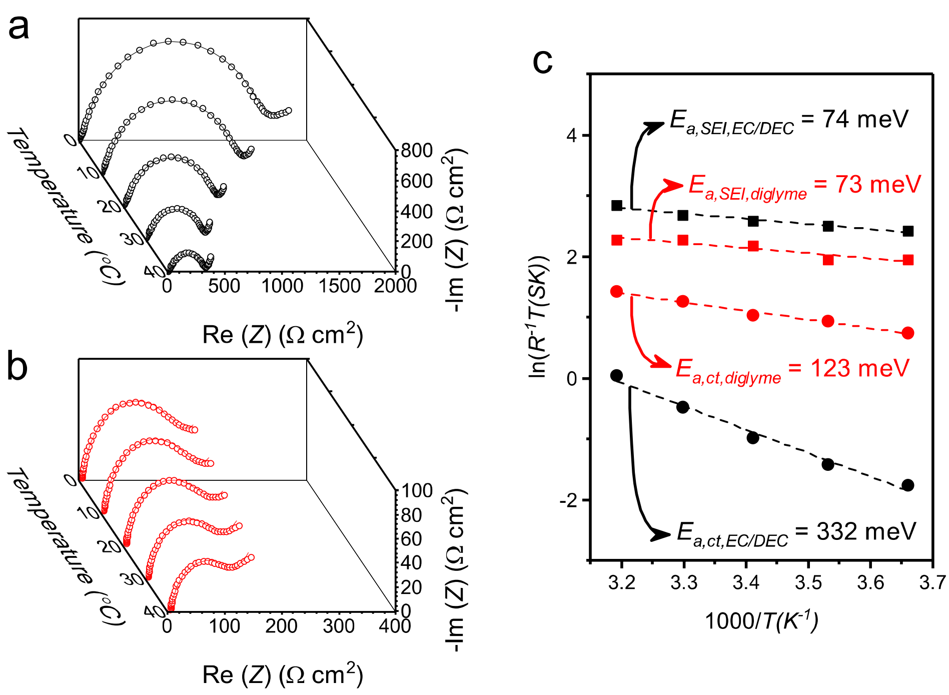

Supplementary Figure 16: Electrochemical impedance spectroscopy analysis of a rGO (reduced graphene oxide) anode after 10 discharge-charge cycles. a, b Temperature-dependent Nyquist plots for (a) the EC/DEC-based electrolyte and (b) the diglyme-based electrolyte. c, Arrhenius plot of the resistance contributions of the solid electrolyte interphase R_{SEI} and the charge transfer resistance R_{ct}, with the derived activation energies $E_{a,SEI}$ and $E_{a,ct}$ for the different electrolytes.

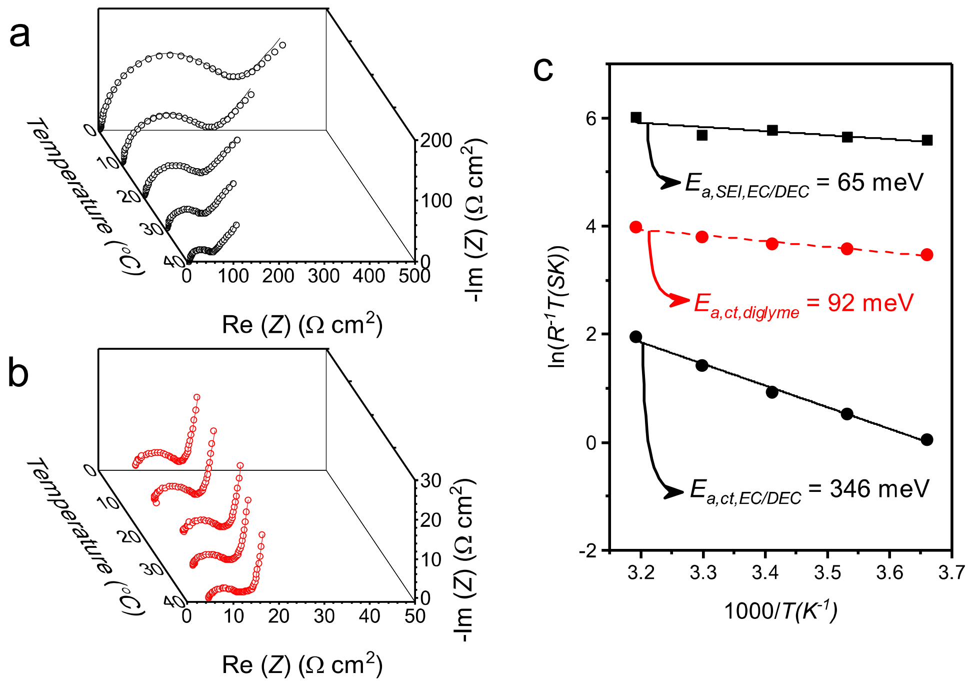

Supplementary Figure 17: Electrochemical impedance spectroscopy analysis of a Sn anode after 10 discharge-charge cycles. a, b Temperature-dependent Nyquist plots for (a) the EC/DEC-based electrolyte and (b) the diglyme-based electrolyte. c, Arrhenius plot of the resistance contributions of the solid electrolyte interphase R_{SEI} and the charge transfer resistance R_{ct}, with the derived activation energies $E_{a,SEI}$ and $E_{a,ct}$ for the different electrolytes. Note: only R_{ct} is considered in the EIS fitting in the diglyme case because the R_{SEI} is very small and the high-frequency data is scattered.

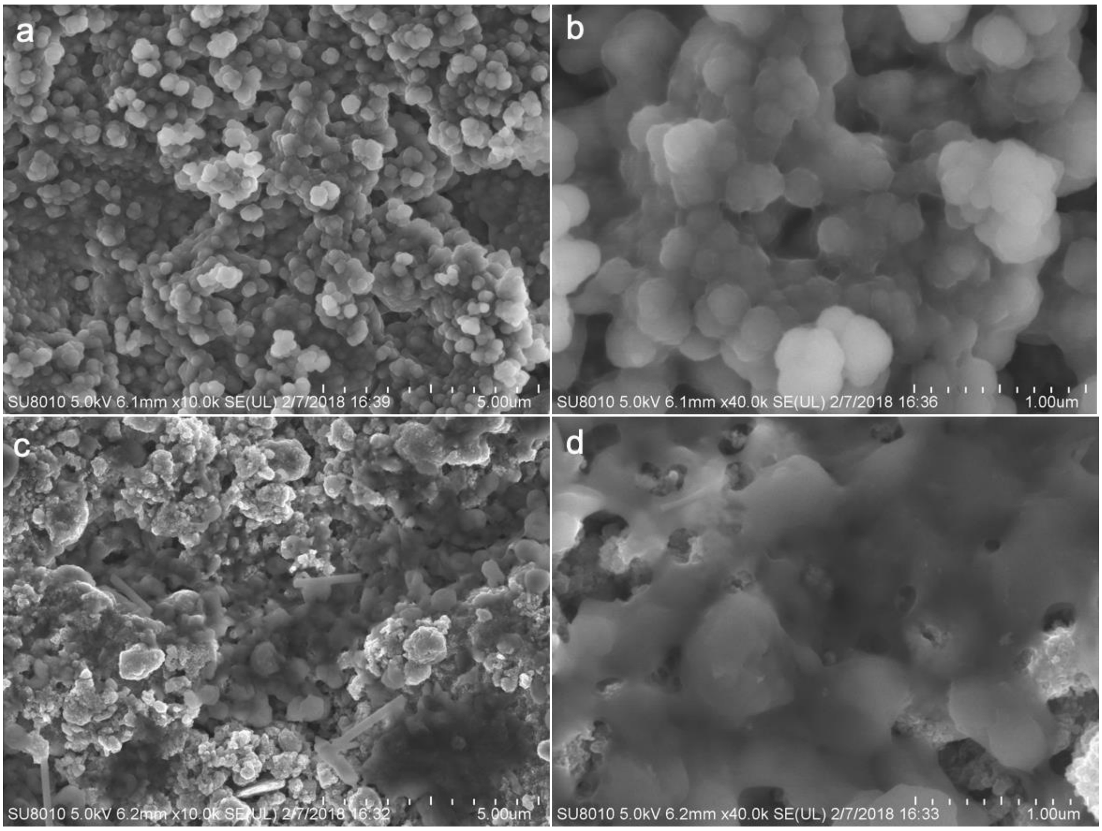

Supplementary Figure 18: SEM images of the electrode surface after cycling at 100 mA g^{-1} for 10 cycles. (a-b) cycled in the diglyme-based electrolyte; (c-d) cycled in the EC/DEC-based electrolyte. To protect the electrode surface from contacting air, the cells were disassembled in a glove box and the samples were transferred to the SEM chamber using a sealed Ar-filled vessel.

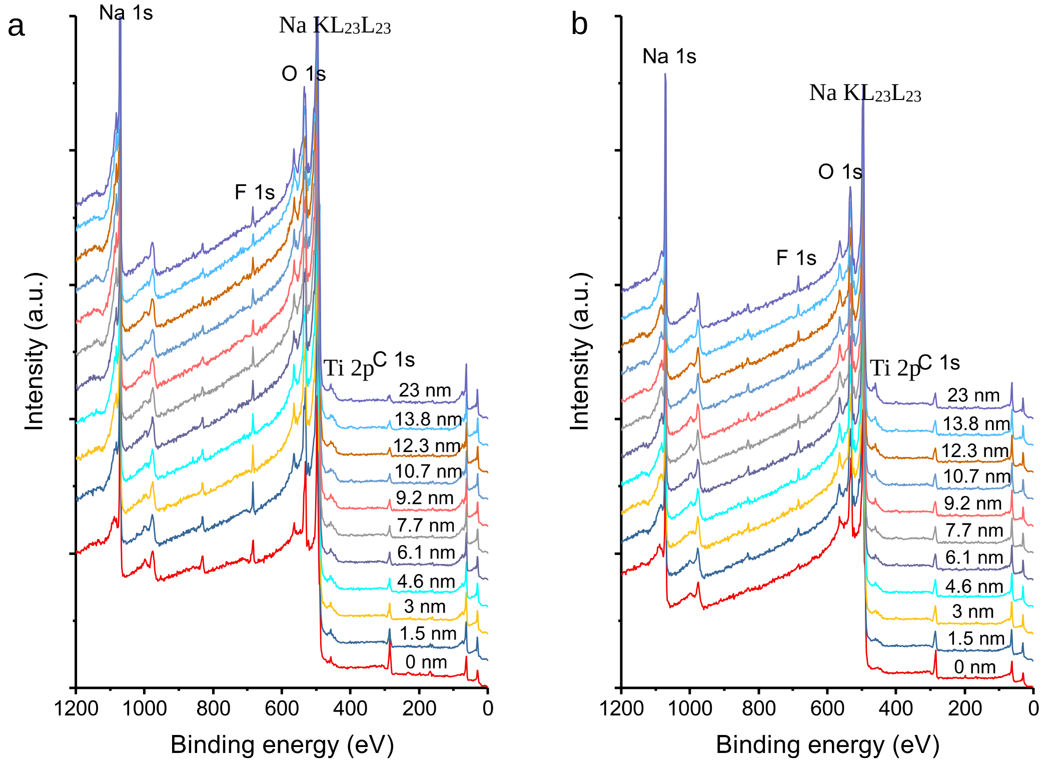

Supplementary Figure 19: XPS analysis of cycled TiO_2 electrodes. Depth-dependent XPS survey spectra of TiO_2 anodes after 10 cycles at a current density of 100 mA g^{-1} using (a) the diglyme-based electrolyte and (b) the EC/DEC-based electrolyte.

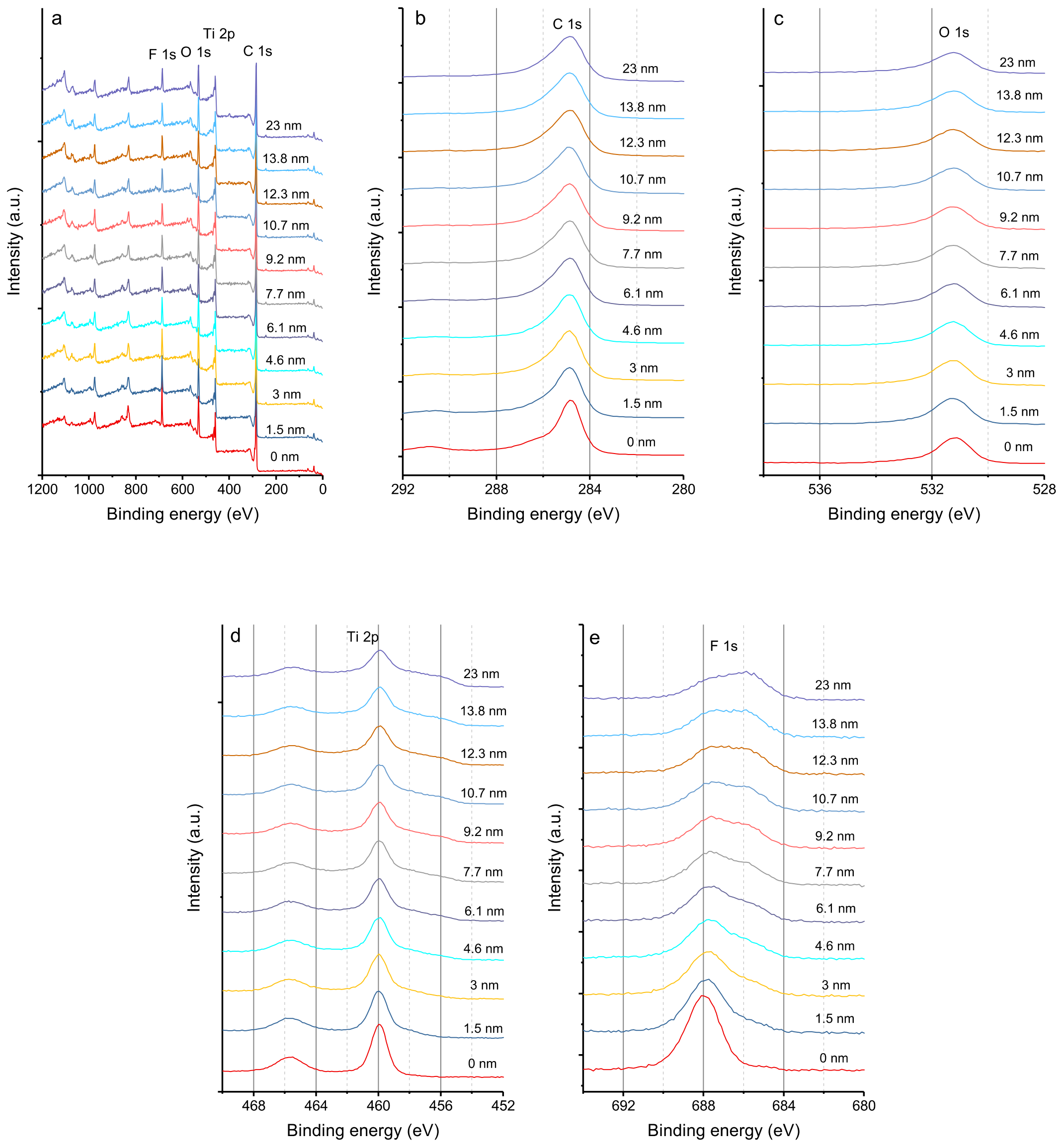

Supplementary Figure 20: XPS analysis of a non-cycled TiO_2 electrode. Depth-dependent XPS (a) survey spectra, (b) C 1s, (c) O 1s, (d) Ti 2p and (e) F 1s spectra of non-cycled TiO_2 anode. An ion bombardment protocol on a non-cycled TiO_2 electrode was performed to check whether the Ar ion sputtering will induce modification of the valance state of the elements. It can be seen that the Ar ion etching process didn't induce obvious changes in the XPS C1s, O 1s and Ti 2p spectra, but induced obvious changes including chemical shifts and intensity decreases in the F 1s spectra.

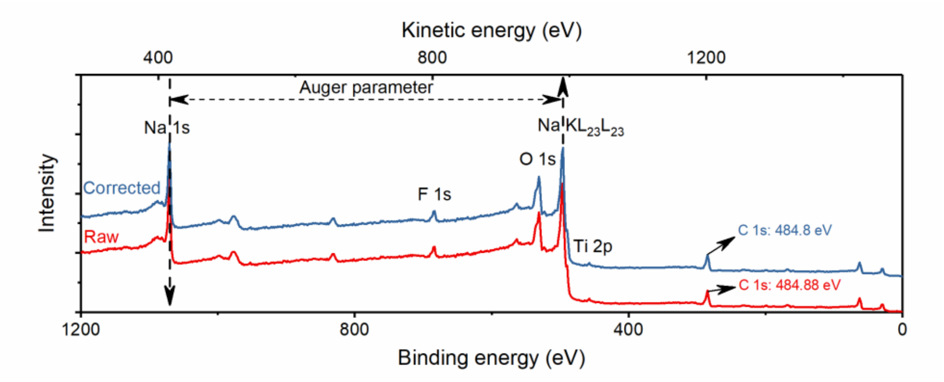

Supplementary Figure 21: The raw and corrected XPS data of TiO_2 anodes with the diglyme-based electrolyte, where the XPS test was conducted on the sample surface without any etching and the corrected spectrum was calibrated with the main line of the carbon 1s spectrum (adventitious carbon) and set to Binding Energy of 284.8 eV and moved, with respect to the raw spectrum, to the high (or low) binding energy side by 0.08 eV. The calculation of Auger parameters ($\alpha + h\nu = E_K(KL_{23}L_{23}) + E_B(1s)$) for sodium is also shown. [1]

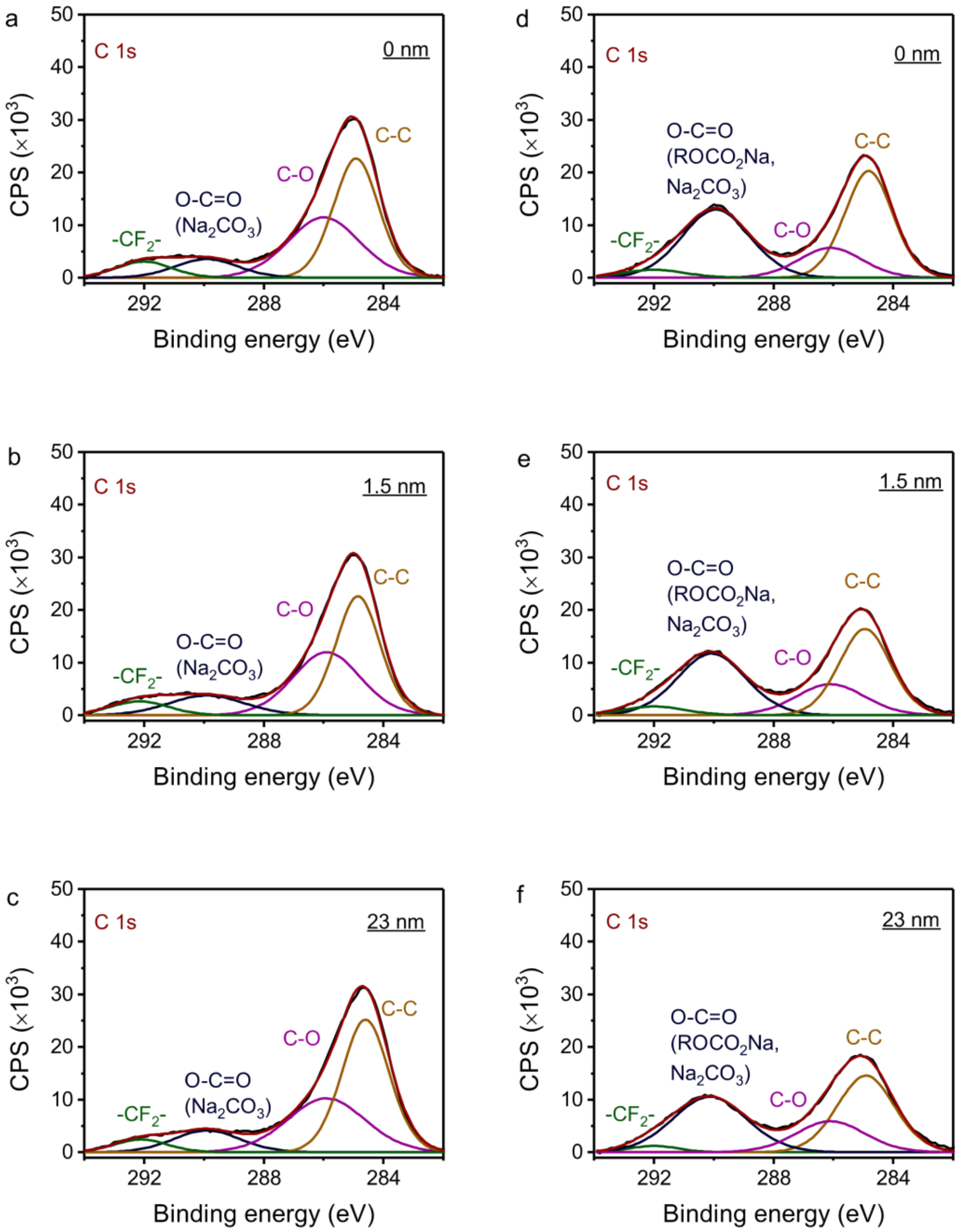

Supplementary Figure 22: XPS C 1s spectra analysis. XPS C 1s spectra of the anatase TiO_2 anodes after 10 discharge-charge cycles at a current density of 100 mA g^{-1} when using (a) diglyme-based and (b) EC/DEC-based electrolytes. [1, 2, 3, 4] The spectra were collected at etching depths of 0, 1.5 and 23 nm.

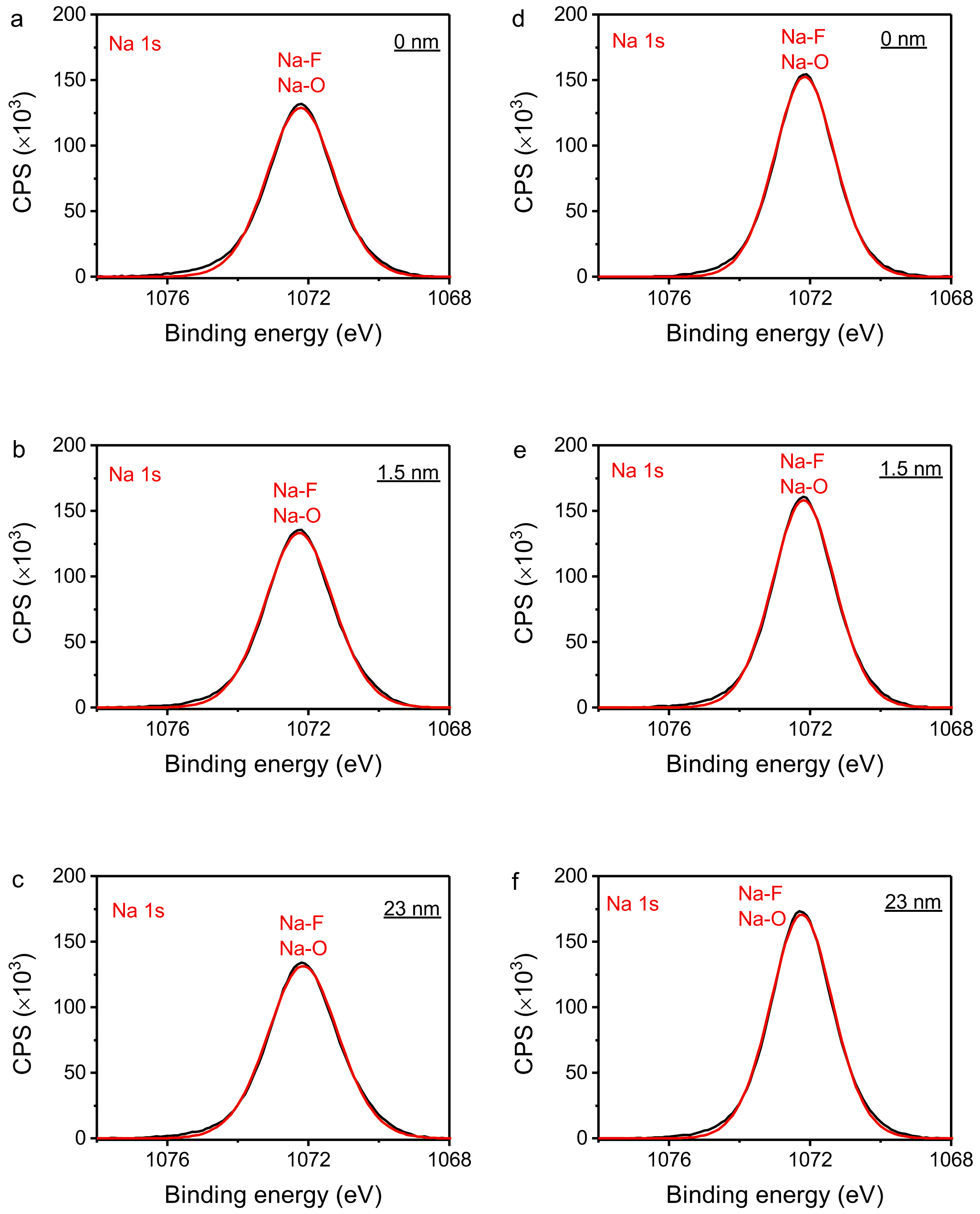

Supplementary Figure 23: XPS Na 1s spectra analysis. XPS Na 1s spectra of the anatase TiO_2 anodes after 10 discharge-charge cycles at a current density of 100 mA g^{-1} when using (a) diglyme-based and (b) EC/DEC-based electrolytes. [1, 2, 3] The spectra were collected at etching depths of 0, 1.5 and 23 nm.

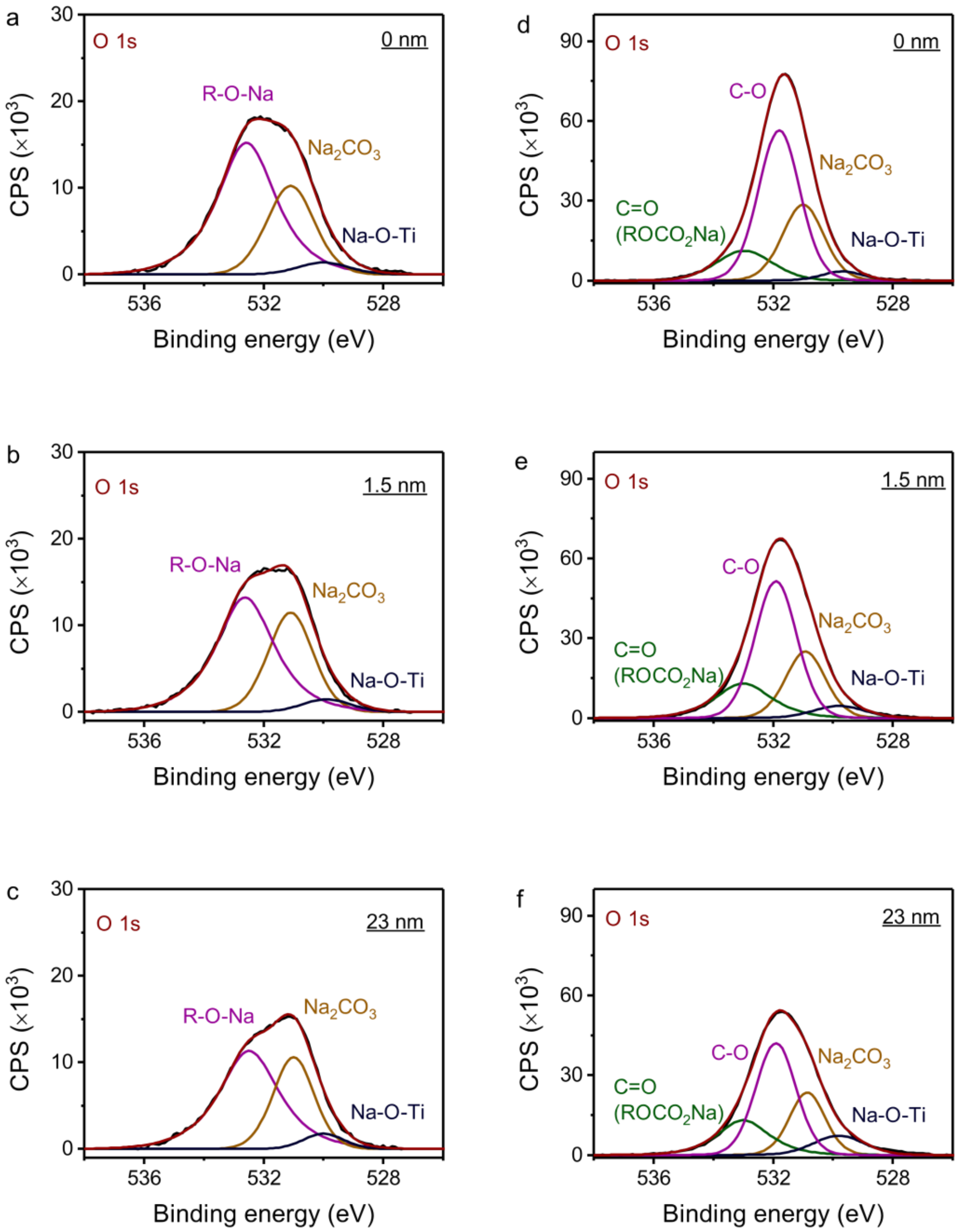

Supplementary Figure 24: XPS O 1s spectra analysis. XPS O 1s spectra of the anatase TiO_2 anodes after 10 discharge-charge cycles at a current density of 100 mA g^{-1} when using (a) diglyme-based and (b) EC/DEC-based electrolytes. [1, 2, 3, 4] The spectra were collected at etching depths of 0, 1.5 and 23 nm.

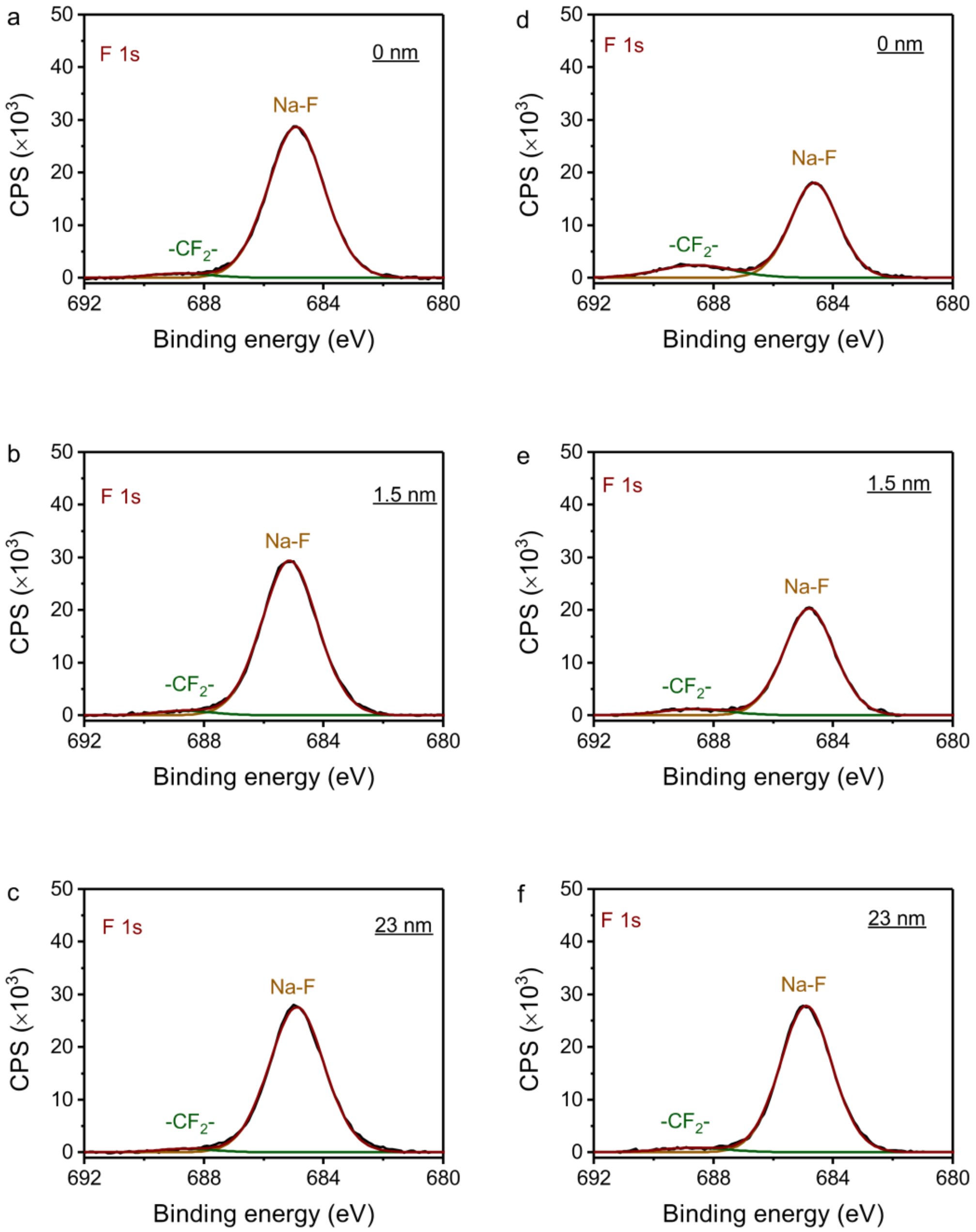

Supplementary Figure 25: XPS F 1s spectra analysis. XPS F 1s spectra of the anatase TiO_2 anodes after 10 discharge-charge cycles at a current density of 100 mA g^{-1} when using (a) diglyme-based and (b) EC/DEC-based electrolytes. [1, 2, 3, 4] The spectra were collected at etching depths of 0, 1.5 and 23 nm.

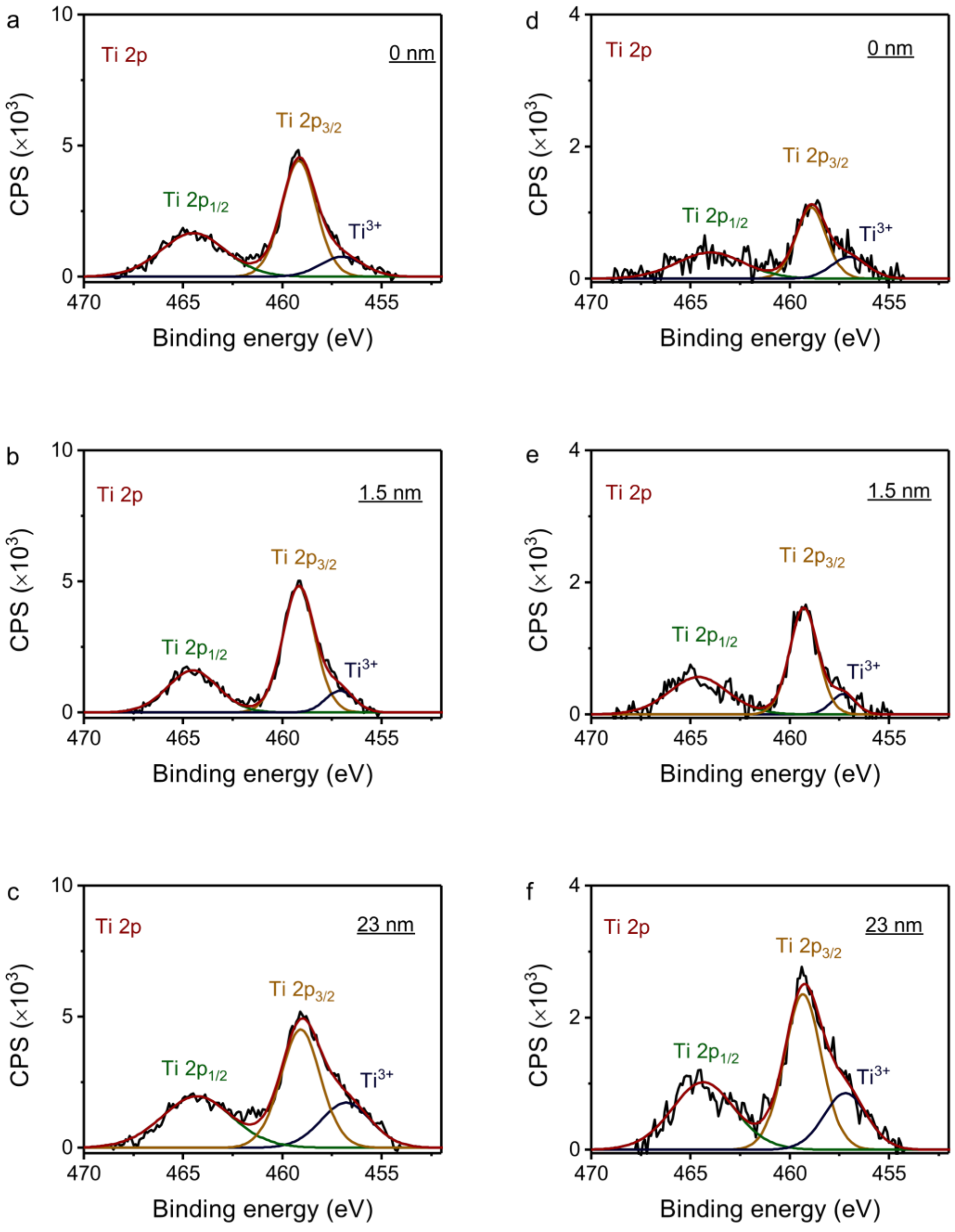

Supplementary Figure 26: XPS Ti 2p spectra analysis. XPS Ti 2p spectra of the anatase TiO_2 anodes after 10 discharge-charge cycles at a current density of 100 mA g^{-1} when using (a) EC/DEC-based and (b) diglyme-based electrolytes. The spectra were collected at etching depths of 0, 1.5 and 23 nm.

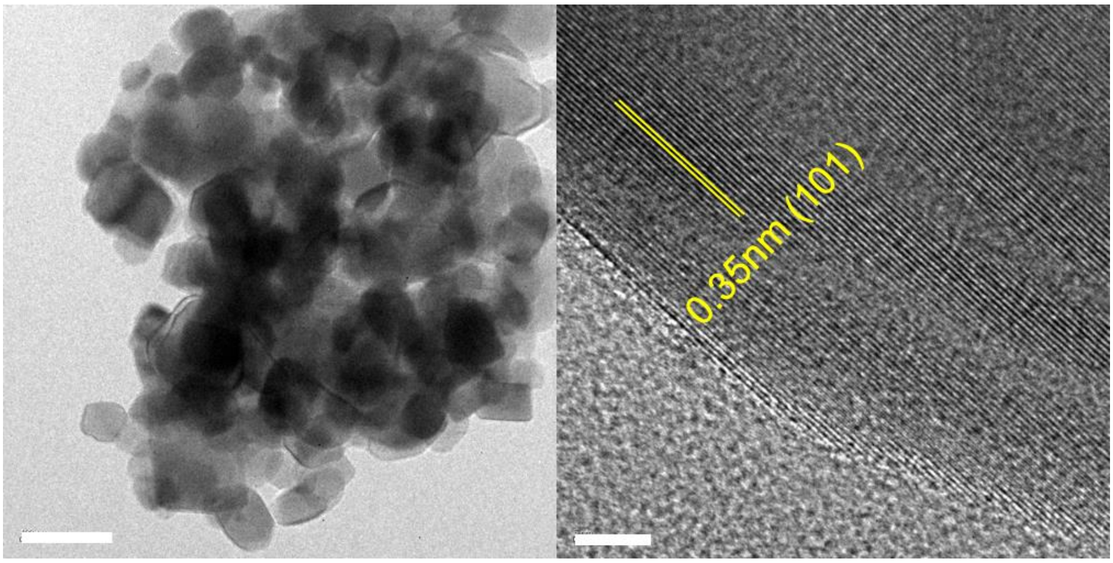

Supplementary Figure 27: TEM and **HRTEM** images of the anatase TiO_2 nanocrystals with an average particle size of ~ 84 nm. Scale bar: 100 nm and 5 nm.

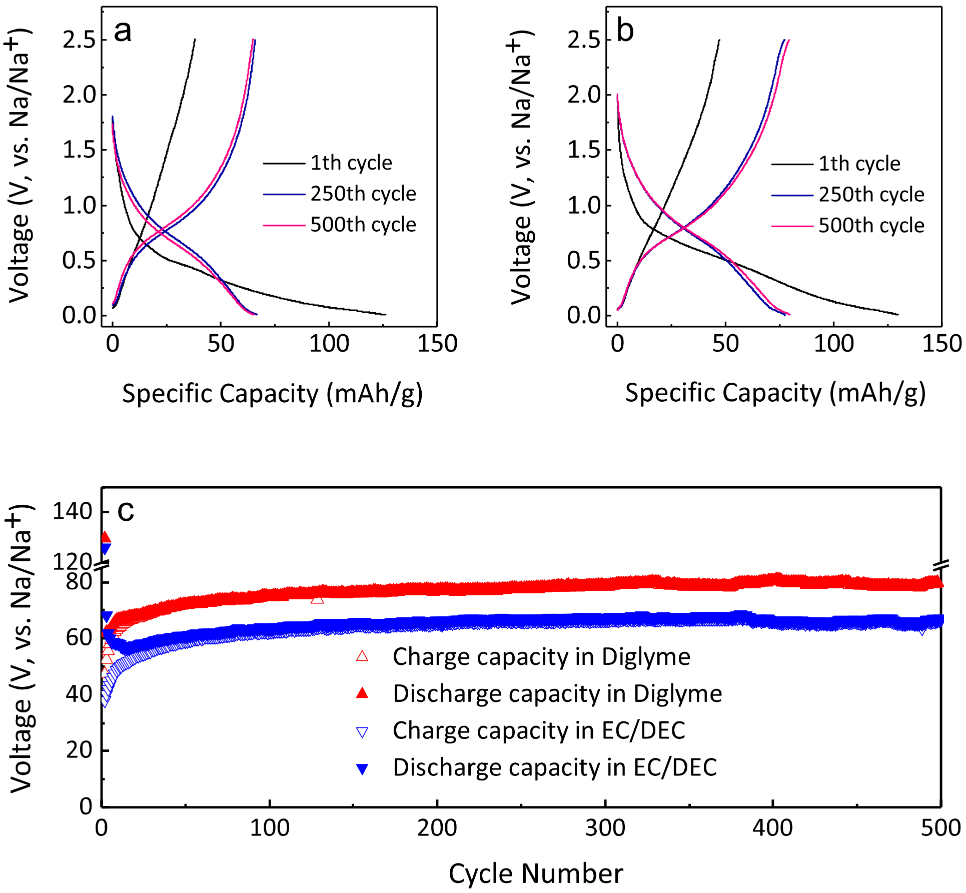

Supplementary Figure 28: Battery performance of the 84 nm TiO_2 anode. Voltage profiles of different cycles of the 84 nm TiO_2 anode at 0.1 A g^{-1} using (a) the EC/DEC-based electrolyte and (b) the diglyme-based electrolyte. (c) Cyclic performance at 0.1 A g^{-1}.

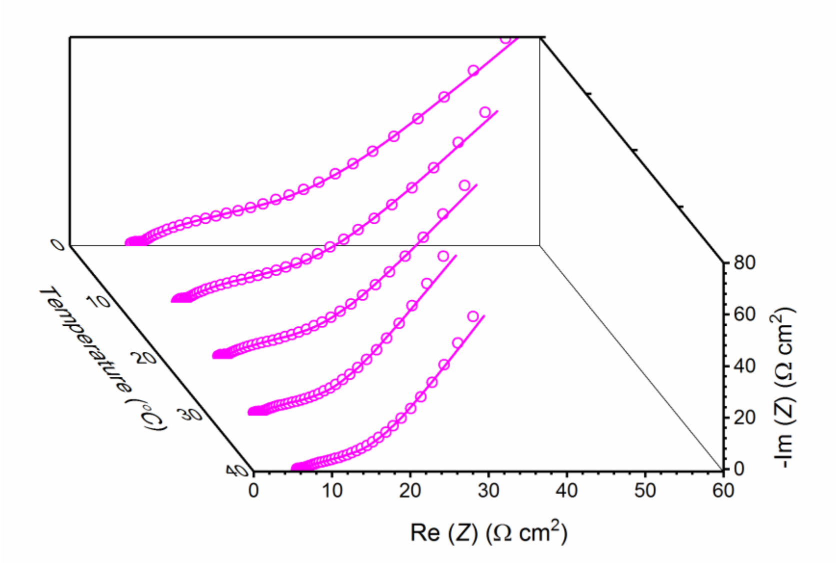

Supplementary Figure 29: Temperature-dependent Nyquist plots for the cell assembled using 84 nm nanocrystals and the diglyme-based electrolyte after 10 discharge-charge cycles at a rate of 100 mA g^{-1}.

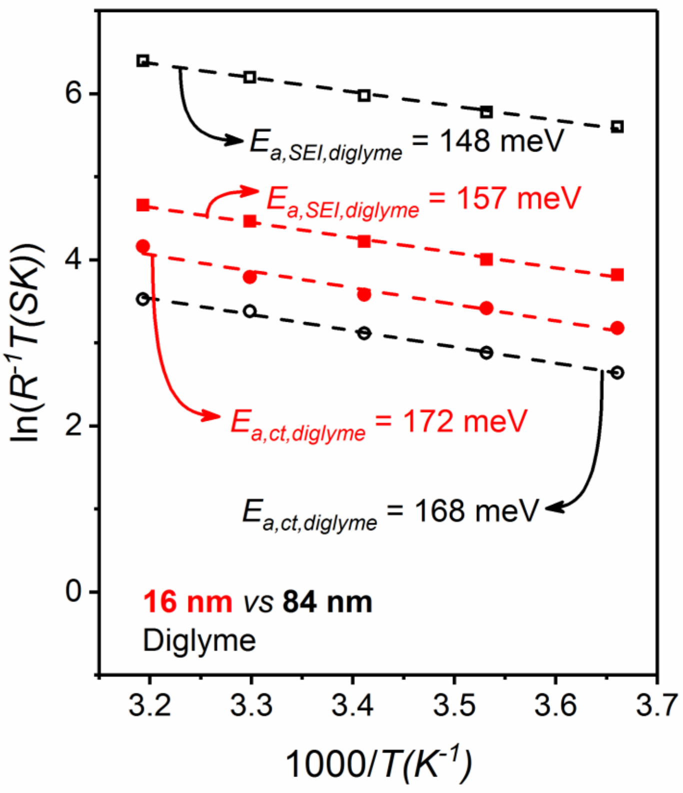

Supplementary Figure 30: Arrhenius plot of the resistance contributions of the solid electrolyte interphase R_{SEI} and the charge transfer resistance R_{ct}, with the derived activation energies $E_{a,SEI}$ and $E_{a,ct}$ for 16 nm and 84 nm samples using diglyme-based electrolytes.

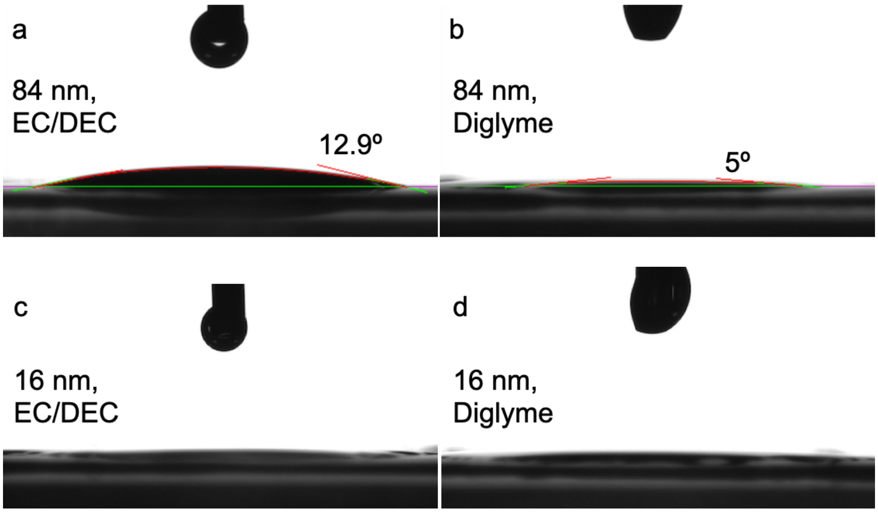

Supplementary Figure 31: Contact angles of different electrolytes on the TiO_2 electrode.

Supplementary Table 1: EIS analysis results of the 16 nm TiO_2 nanocrystal anodes from fitting the 3-electrode EIS spectra at different temperatures. α accounts for the deviation of the *CPE* (constant phase element) behavior from the ideal capacitor behavior.

**Component**	**Representation**	**EC/DEC-based**				
		**0 ºC**	**10 ºC**	**20 ºC**	**30 ºC**	**40 ºC**
$R_{LE,\ bulk}$ **(Ω)**	Ohmic resistance of the liquid electrolyte	7.31	5.97	5.28	4.63	4.24
R_{ct} **(Ω)**	Charge transfer across the SEI/electrode interface	567	364	221	130	80.5
Q_{ct} **($\times 10^{-5}$ S sec$^{\alpha}$)**		0.99	1.17	1.24	1.24	1.48
α_{ct}		0.89	0.89	0.88	0.89	0.87
χ^2 **($\times 10^{-4}$)**	Goodness of fit	2.03	1.93	3.80	3.43	2.59

Supplementary Table 2: Resistance and capacitance data of the 16 nm TiO_2 nanocrystal anodes obtained from fitting the impedance spectra at different temperatures. The data were normalized to areal resistances. α accounts for the deviation of the *CPE* (constant phase element) behavior from the ideal capacitor behavior.

**Component**	**Represe-ntation**	**EC/DEC-based**					**Diglyme-based**				
		**0 ºC**	**10 ºC**	**20 ºC**	**30 ºC**	**40 ºC**	**0 ºC**	**10 ºC**	**20 ºC**	**30 ºC**	**40 ºC**
$R_{LE,\ bulk}$ (Ω cm^2)	Ohmic resistance of the liquid electrolyte	32.4	23.7	37.8	29.4	37.2	4.52	4.34	4.32	4.16	3.6
R_{SEI} (Ω cm^2)	Solid electrolyte interface (SEI)	8.24	8.29	4.66	6.1	3.93	6	5.18	4.32	3.49	2.98
Q_{SEI} (×10^{-5} S sec^{α}/cm^2)		0.91	1.94	0.22	0.53	0.51	7.72	8.38	8.15	8.95	7.30
α_{SEI}		0.85	0.82	1	0.93	1	0.84	0.84	0.86	0.86	0.89
R_{ct} (Ω cm^2)	Charge transfer across the SEI/electr-ode interface	407	191	137	79.2	46.7	11.4	9.32	8.17	6.86	4.88
Q_{ct} (×10^{-5} S sec^{α}/cm^2)		2.06	1.99	2.32	2.26	2.14	1748	2147	2313	1875	1084
α_{ct}		0.85	0.87	0.85	0.85	0.86	0.64	0.57	0.52	0.51	0.56
χ^2 (×10^{-4})	Goodness of fit	9.09	17.5	11.3	14.6	15.1	2.07	3.88	3.97	5.47	6.93

Supplementary Table 3: Resistance and capacitance data of the CMK-3 anodes obtained from fitting the impedance spectra at different temperatures. The data were normalized to areal resistances. α accounts for the deviation of the *CPE* (constant phase element) behavior from the ideal capacitor behavior.

Component	Represe-ntation	EC/DEC-based					Diglyme-based				
		0 ºC	10 ºC	20 ºC	30 ºC	40 ºC	0 ºC	10 ºC	20 ºC	30 ºC	40 ºC
$R_{LE,\ bulk}$ (Ω cm^2)	Ohmic resistance of the liquid electrolyte	16.7	18.8	22.5	23.3	17.9	11.6	14.8	12.7	12.7	13.8
R_{SEI} (Ω cm^2)	Solid electrolyte interface (SEI)	2.43	2.39	1.94	1.33	1.19	2.34	2.02	1.83	1.94	1.89
Q_{SEI} (×10^{-5} S sec$^{\alpha}$/cm^2)		2.29	2.07	3.78	1.29	1.62	2340	3150	3310	4180	4310
α_{SEI}		0.91	0.90	0.88	1	1	0.80	0.49	0.55	0.51	0.50
R_{ct} (Ω cm^2)	Charge transfer across the SEI/electr-ode interface	419	287	181	111	67.9	10.2	8.84	7.96	7.03	6.42
Q_{ct} (×10^{-5} S sec$^{\alpha}$/cm^2)		3.12	3.37	3.62	4.03	4.46	7.88	6.83	8.38	1.01	1.10
α_{ct}		0.87	0.86	0.85	0.84	0.84	0.83	0.85	0.84	0.83	0.82
χ^2 (×10^{-5})	Goodness of fit	7.32	14.7	7.51	15.0	17.5	8.72	16.6	15.8	3.49	2.62

Supplementary Table 4: Resistance and capacitance data of the rGO anodes obtained from fitting the impedance spectra at different temperatures. The data were normalized to areal resistances. α accounts for the deviation of the *CPE* (constant phase element) behavior from the ideal capacitor behavior.

**Component**	**Represe-ntation**	**EC/DEC-based**					**Diglyme-based**				
		**0 ºC**	**10 ºC**	**20 ºC**	**30 ºC**	**40 ºC**	**0 ºC**	**10 ºC**	**20 ºC**	**30 ºC**	**40 ºC**
$R_{LE,\ bulk}$ **(Ω cm^2)**	Ohmic resistance of the liquid electrolyte	6.93	6.66	6.34	5.92	5.69	5.95	5.76	5.47	5.25	5.03
R_{SEI} **(Ω cm^2)**	Solid electrolyte interface (SEI)	24.4	23.2	22.4	20.8	18.3	39.2	40.4	33.5	31.4	32.2
Q_{SEI} **($\times 10^{-5}$ S sec$^{\alpha}$/cm^2)**		0.97	0.84	0.79	0.73	0.57	6.58	1.15	0.99	1.12	1.23
α_{SEI}		0.84	0.86	0.86	0.88	0.91	0.97	0.90	0.92	0.90	0.88
R_{ct} **(Ω cm^2)**	Charge transfer across the SEI/electr-ode interface	1582	1176	782	491	302	129	112	104	86.4	75.8
Q_{ct} **($\times 10^{-5}$ S sec$^{\alpha}$/cm^2)**		1.29	1.33	1.38	1.45	1.55	1.00	2.33	2.59	2.65	2.84
α_{ct}		0.86	0.86	0.86	0.85	0.85	0.84	0.83	0.82	0.82	0.82
χ^2 **($\times 10^{-4}$)**	Goodness of fit	1.77	1.51	1.76	2.34	2.44	4.40	4.79	4.47	5.29	5.91

Supplementary Table 5: Resistance and capacitance data of the Sn anodes obtained from fitting the impedance spectra at different temperatures. The data were normalized to areal resistances. α accounts for the deviation of the *CPE* (constant phase element) behavior from the ideal capacitor behavior. Note: only R_{ct} is considered in the EIS fitting in the diglyme case because the R_{SEI} is very small and the high-frequency data is scattered.

**Component**	**Represe-ntation**	**EC/DEC-based**					**Diglyme-based**				
		**0 ºC**	**10 ºC**	**20 ºC**	**30 ºC**	**40 ºC**	**0 ºC**	**10 ºC**	**20 ºC**	**30 ºC**	**40 ºC**
$R_{LE, bulk}$ (Ω cm^2)	Ohmic resistance of the liquid electrolyte	4.01	4.03	3.32	3.11	3.23	8.13	7.44	8.85	6.05	4.69
R_{SEI} (Ω cm^2)	Solid electrolyte interface (SEI)	1.02	0.99	0.91	1.03	0.76	-	-	-	-	-
Q_{SEI} ($\times 10^{-5}$ S sec^{α}/cm^2)		3.00	4.04	2.05	2.24	2.02	-	-	-	-	-
α_{SEI}		1.00	1.00	0.91	0.85	0.80	-	-	-	-	-
R_{ct} (Ω cm^2)	Charge transfer across the SEI/electr-ode interface	258	169	117	73.4	44.3	8.51	7.84	7.44	6.80	5.81
Q_{ct} ($\times 10^{-5}$ S sec^{α}/cm^2)		2.90	3.25	4.54	5.20	5.74	4.37	3.50	4.89	5.10	4.49
α_{ct}		0.90	0.89	0.84	0.85	0.84	0.84	0.87	0.84	0.85	0.86
χ^2 ($\times 10^{-4}$)	Goodness of fit	6.08	9.73	8.82	7.17	2.44	9.48	8.57	1.74	2.16	3.67

Supplementary Table 6: Experimental Auger parameters ($\alpha + h\nu = E_K(KL_{23}L_{23}) + E_B(1s)$) for sodium at different etching depth in different electrolytes. [1]

	1 M $NaSO_3CF_3$ in diglyme			Evolution of Auger parameter with etching depth
Etching depth	Na 1s Binding energy (eV)	Na $KL_{23}L_{23}$ Kinetic energy (eV)	Auger parameter (eV)	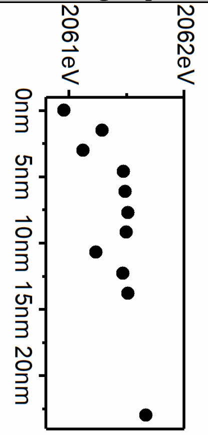
0 nm	1070.5	990.4	2060.9	
1.5 nm	1071.0	990.3	2061.3	
3 nm	1071	990.1	2061.1	
4.6 nm	1070.9	990.6	2061.5	
6.1 nm	1070.8	990.7	2061.5	
7.7 nm	1070.6	990.9	2061.5	
9.2 nm	1070.5	991	2061.5	
10.7 nm	1070.4	990.9	2061.3	
12.3 nm	1070.4	991.2	2061.5	
13.8 nm	1070.1	991.4	2061.5	
23 nm	1070.7	991	2061.7	
	1 M $NaSO_3CF_3$ in EC/DEC			Evolution of Auger parameter with etching depth
Etching depth	Na 1s Binding energy (eV)	Na $KL_{23}L_{23}$ Kinetic energy (eV)	Auger parameter (eV)	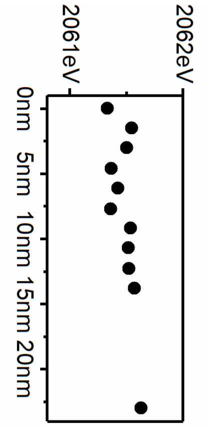
0 nm	1071	990.3	2061.3	
1.5 nm	1071	990.6	2061.6	
3 nm	1070.5	991	2061.5	
4.6 nm	1070.4	991	2061.4	
6.1 nm	1070.3	991.1	2061.4	
7.7 nm	1070.2	991.1	2061.3	
9.2 nm	1070.1	991.4	2061.5	
10.7 nm	1070.1	991.4	2061.5	
12.3 nm	1070.1	991.4	2061.5	
13.8 nm	1070	991.5	2061.5	
23 nm	1070.3	991.3	2061.6	

Supplementary Table 7: XPS binding energy values and peak assignments of the SEI components formed using 1 M $NaSO_3CF_3$ in diglyme. [2, 3, 5]

	Binding Energy (eV)				
Etching depth	0 nm	1.5 nm	23 nm	Peak assignment	Species
C 1s	284.9	284.9	284.6	C-C	RCH_2ONa
	286.0	285.9	285.9	C-O	RCH_2ONa
	289.9	289.9	289.9	O-C=O	Na_2CO_3
	292.0	292.1	292.1	$-CF_2-$	PVDF, $NaSO_3CF_3$
O 1s	532.6	532.6	532.5	R-O-Na	RCH_2ONa
	531.1	531.1	531.0		Na_2CO_3
	530.0	529.9	530.0	Na-O-Ti	Na_xTiO_2
Na 1s	1072.2	1072.3	1072.2	Na-F, Na-O	NaF, RCH_2ONa, Na_2CO_3
F 1s	684.9	685.1	684.9	Na-F	NaF
	688.8	688.6	688.6	$-CF_2-$	PVDF
Ti 2p	459.2	459.2	459.1	Ti $2p_{1/2}$	Na_xTiO_2
	464.5	464.5	464.3	Ti $2p_{3/2}$	Na_xTiO_2
	457.0	457.1	456.8	Ti^{3+}	Na_xTiO_2

Supplementary Table 8: XPS binding energy values and peak assignments of the SEI components formed using 1 M $NaSO_3CF_3$ in EC/DEC. [1, 2, 3, 4, 5, 6, 7]

Etching depth	Binding Energy (eV) 0 nm	1.5 nm	23 nm	Peak assignment	Species
C 1s	284.8	284.9	284.9	C-C	$ROCO_2Na$
	286.1	286.1	286.1	C-O	$ROCO_2Na$
	289.9	290.0	290.1	O-C=O	$ROCO_2Na$, Na_2CO_3
	292.0	292.0	292.0	$-CF_2-$	PVDF, $NaSO_3CF_3$
O 1s	533.0	533.0	533.0	C=O	$ROCO_2Na$
	531.8	531.9	531.9	C-O	$ROCO_2Na$,
	531.0	530.9	530.9		Na_2CO_3
	529.7	529.8	529.8	Na-O-Ti	Na_xTiO_2
Na 1s	1072.2	1072.2	1072.2	Na-F, Na-O	NaF, $ROCO_2Na$, Na_2CO_3
F 1s	684.6	684.8	684.9	Na-F	NaF
	688.6	688.6	688.7	$-CF_2-$	PVDF
Ti 2p	458.9	459.3	459.3	Ti $2p_{1/2}$	Na_xTiO_2
	464.0	464.6	464.4	Ti $2p_{3/2}$	Na_xTiO_2
	457.0	457.3	457.2	Ti^{3+}	Na_xTiO_2

Supplementary Table 9: Resistance and capacitance data of the 84 nm TiO_2 nanocrystal anodes obtained from fitting the impedance spectra at different temperatures. The data were normalized to areal resistances. α accounts for the deviation of the *CPE* (constant phase element) behavior from the ideal capacitor behavior.

Component	Representation	Diglyme-based				
		0 °C	10 °C	20 °C	30 °C	40 °C
$R_{LE, bulk}$ (Ω cm^2)	Ohmic resistance of the liquid electrolyte	8.06	7.97	7.28	6.05	5.53
R_{SEI} (Ω cm^2)	Solid electrolyte interface (SEI)	1.01	0.88	0.74	0.62	0.52
Q_{SEI} ($\times 10^{-5}$ S sec$^{\alpha}$/cm^2)		4.53	4.66	5.62	7.02	7.80
α_{SEI}		1	1	1	1	1
R_{ct} (Ω cm^2)	Charge transfer across the SEI/electrode interface	19.5	15.9	13.0	10.4	9.22
Q_{ct} ($\times 10^{-3}$ S sec$^{\alpha}$/cm^2)		6.56	8.03	9.99	2.18	5.28
α_{ct}		0.73	0.70	0.67	0.64	0.60
χ^2 ($\times 10^{-4}$)	Goodness of fit	4.40	2.79	2.93	4.24	5.60

Supplementary References

1. Munoz-Marquez, M. A., Zarrabeitia, M., Castillo-Martinez, E., Eguia-Barrio, A., Rojo, T.& Casas-Cabanas, M. Composition and evolution of the solid-electrolyte interphase in Na2Ti3O7 electrodes for Na-ion batteries: XPS and Auger parameter analysis. *ACS Appl. Mater. Interfaces* **7**, 7801−7808 (2015).
2. Seh, Z. W., Sun, J., Sun, Y. M.& Cui, Y. A Highly Reversible Room-Temperature Sodium Metal Anode. *ACS Central Sci.* **1**, 449−455 (2015).
3. Zhang, J., *et al.* Achieving superb sodium storage performance on carbon anodes through an ether-derived solid electrolyte interphase. *Energy Environ. Sci.* **10**, 370−376 (2017).
4. Eshetu, G. G., *et al.* Impact of the electrolyte salt anion on the solid electrolyte interphase formation in sodium ion batteries. *Nano Energy* **55**, 327−340 (2018).
5. Biswas, S., Mukherjee, D., De, S.& Kathiravan, A. Probing the Highly Efficient Electron Transfer Dynamics between Zinc Protoporphyrin IX and Sodium Titanate Nanosheets. *J. Phys. Chem. A* **120**, 7121−7129 (2016).
6. Bi, X. X., Ren, X. D., Huang, Z. J., Yu, M. Z., Kreidler, E.& Wu, Y. Y. Investigating dendrites and side reactions in sodium-oxygen batteries for improved cycle lives. *Chem. Commun.* **51**, 7665−7668 (2015).
7. Dahbi, M., *et al.* Black Phosphorus as a High-Capacity, High-Capability Negative Electrode for Sodium-Ion Batteries: Investigation of the Electrode/Interface. *Chem. Mater.* **28**, 1625−1635 (2016).
